# Supplementary material for: Constrained Dipeptide Surrogates: 5- and 7-Hydroxy Indolizidin-2-one Amino Acid Synthesis from Iodolactonization of Dehydro-2,8-diamino Azelates
Source: Molecules. 2021 Dec 23;27(1):67. doi: 10.3390/molecules27010067 (PMC8746717; doi:10.3390/molecules27010067)
Supplement: Supplementary file 1 [file molecules-27-00067-s001.zip › molecules-1514025-supplementary.pdf]

Constrained Dipeptide Surrogates: 5- and 7- Hydroxy Indolizidin-2-one Synthesis from Iodolactonization of Dehydro-2,8-Diamino Azelate

Ramakotaiah Mulamreddy, [†] William D. Lubell[†]*

[†]Département de Chimie, Université de Montréal, C.P. 6128, Succursale Centre-Ville, Montréal, Québec, H3C3J7, Canada.

Email: William.lubell@umontreal.ca

Supporting Information

Table of contents:

NMR Spectra…………………………………………………………………..……..S02-S35

X-ray data………………………………………………………………………………….S36-S45

¹H NMR, 500 MHz

Solvent: $CDCl_3$

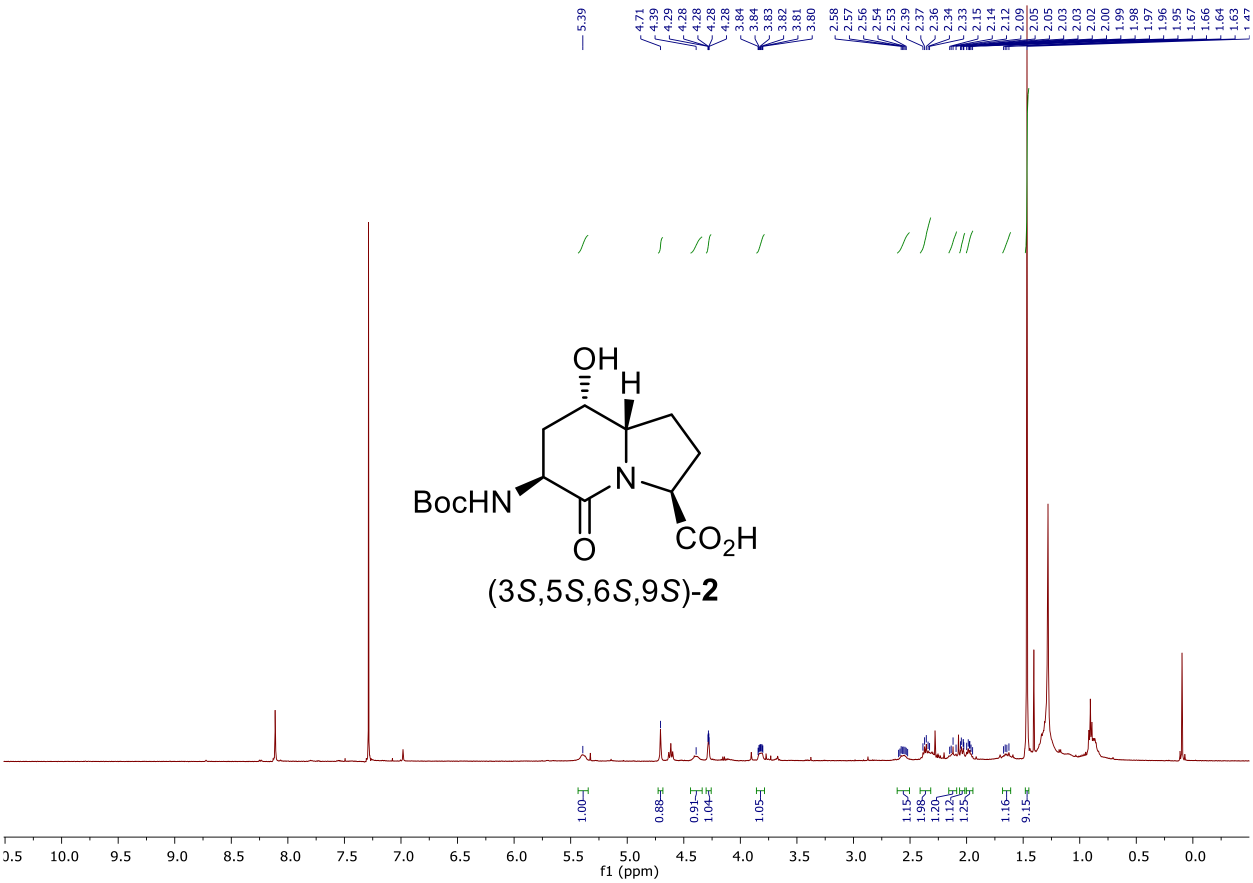

13C NMR, 125 MHz

Solvent: $CDCl_3$

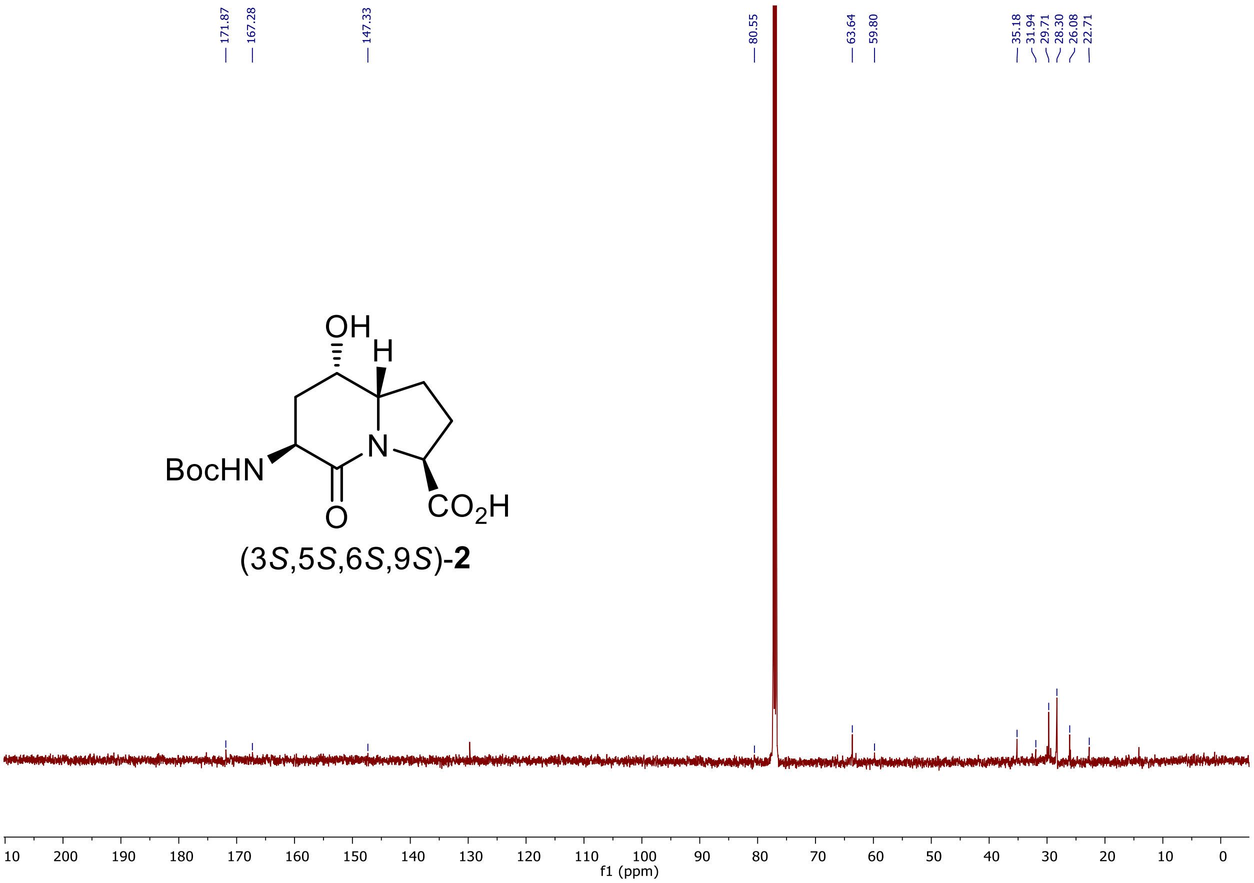

(3*S*,5*S*,6*S*,9*S*)-**2**

^{1}H NMR, 500 MHz

Solvent: CD_3OD

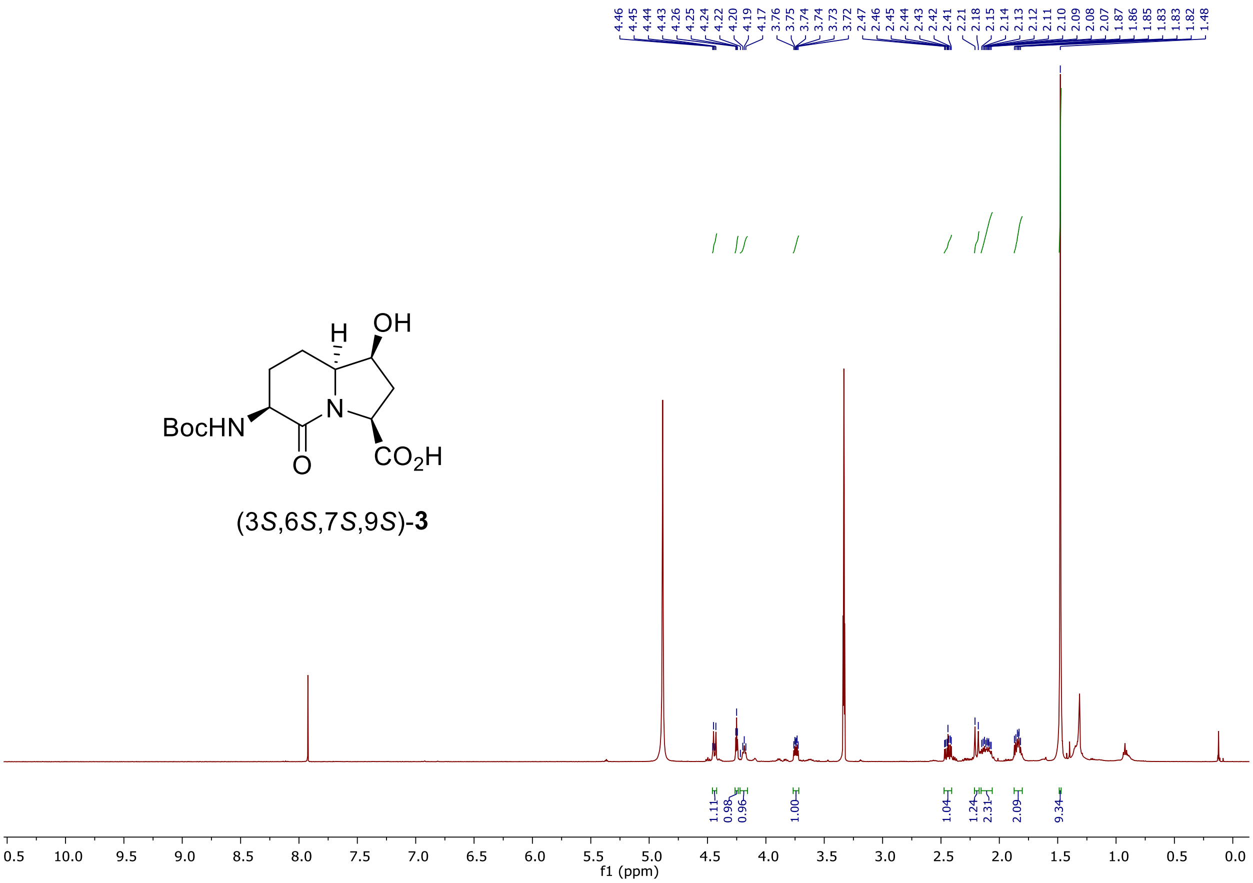

(3*S*,6*S*,7*S*,9*S*)-**3**

13C NMR, 125 MHz

Solvent: CD_3OD

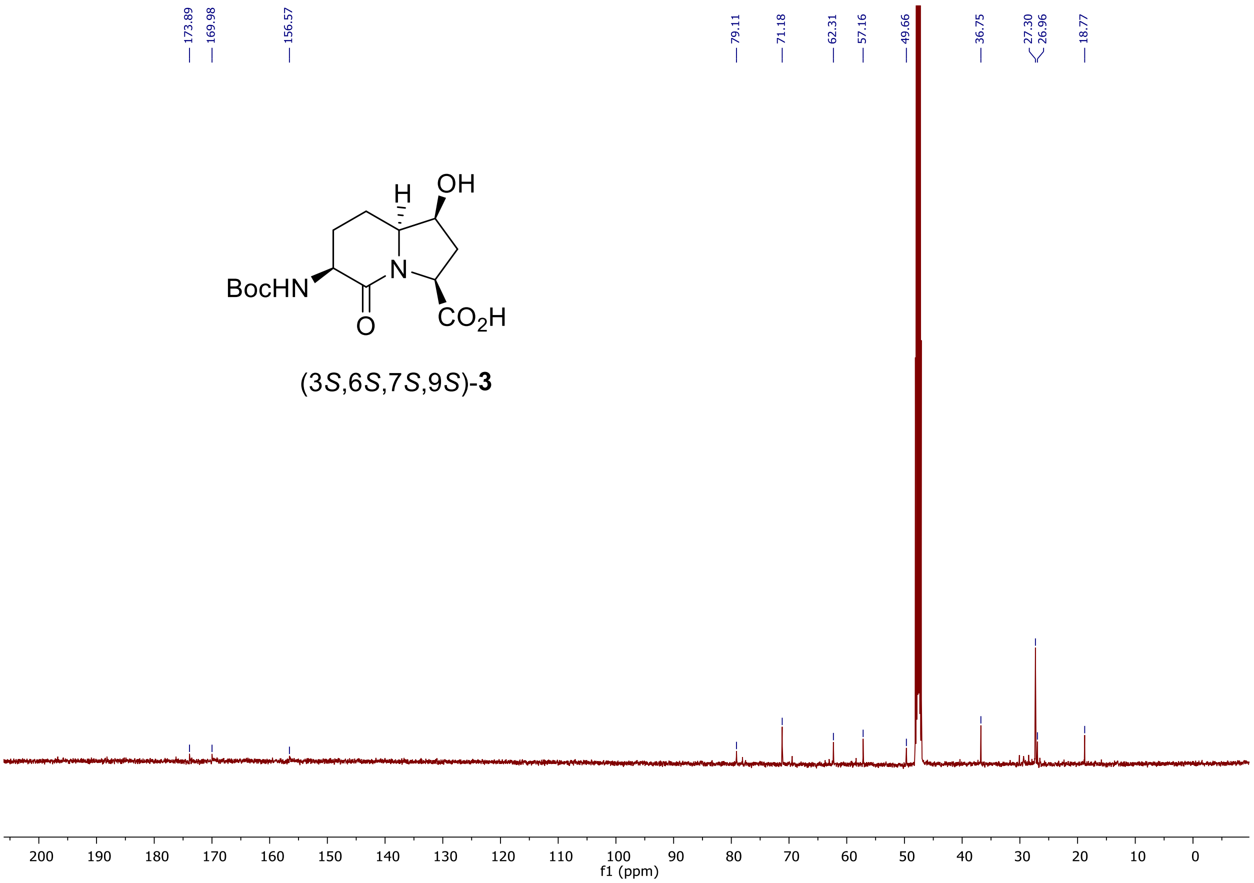

^{1}H NMR, 500 MHz

Solvent: $CDCl_3$

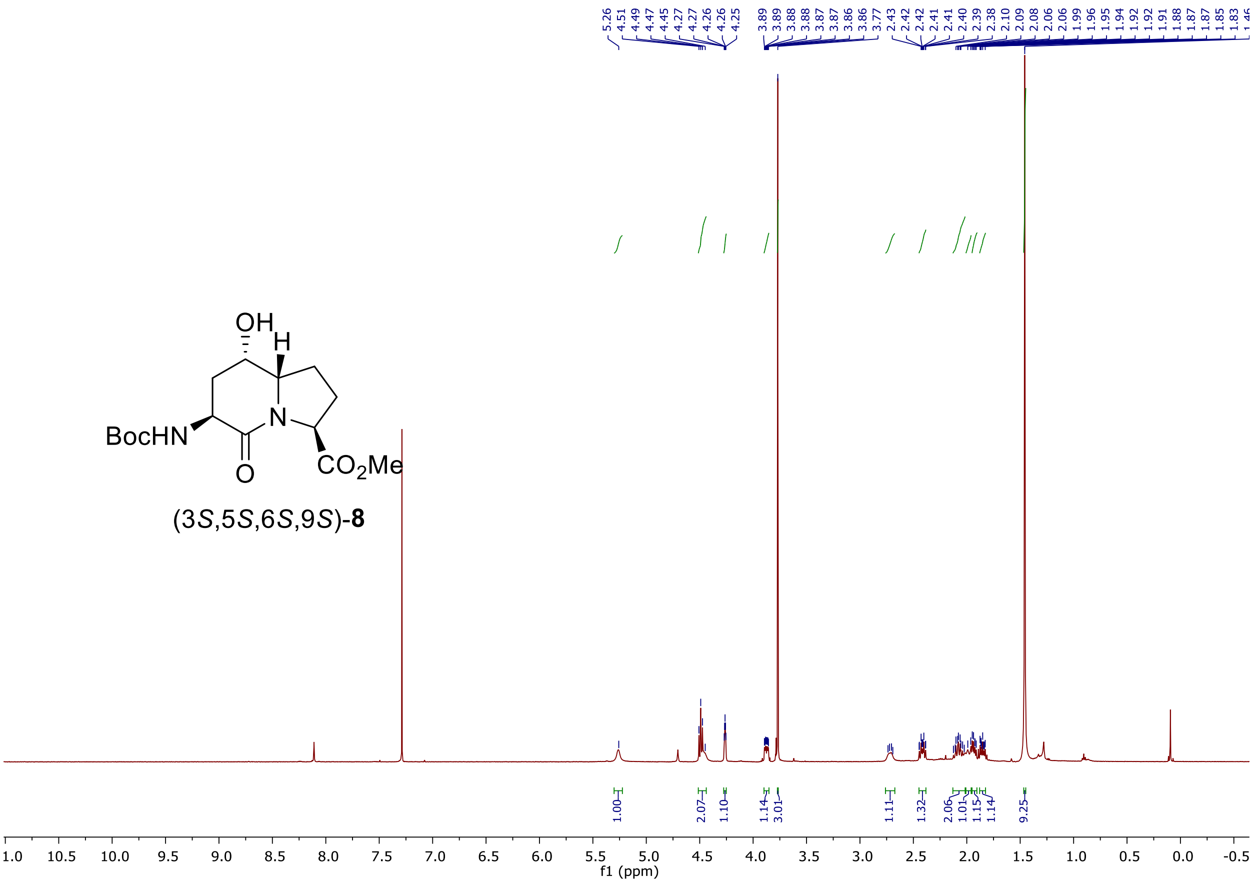

(3*S*,5*S*,6*S*,9*S*)-**8**

13C NMR, 125 MHz

Solvent: CDCl₃

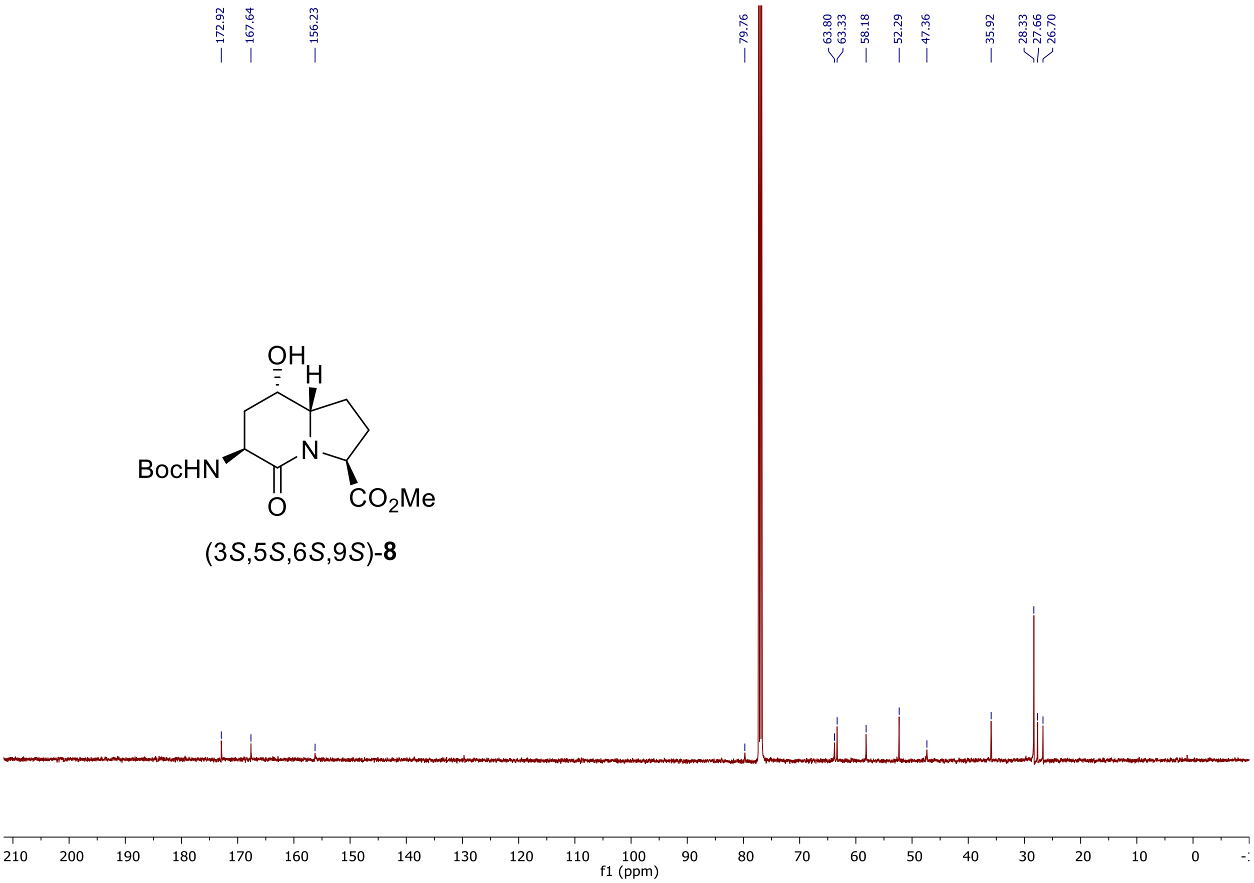

ed-HSQC, 500 MHz

Solvent: $CDCl_3$

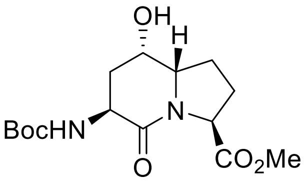

(3*S*,5*S*,6*S*,9*S*)-**8**

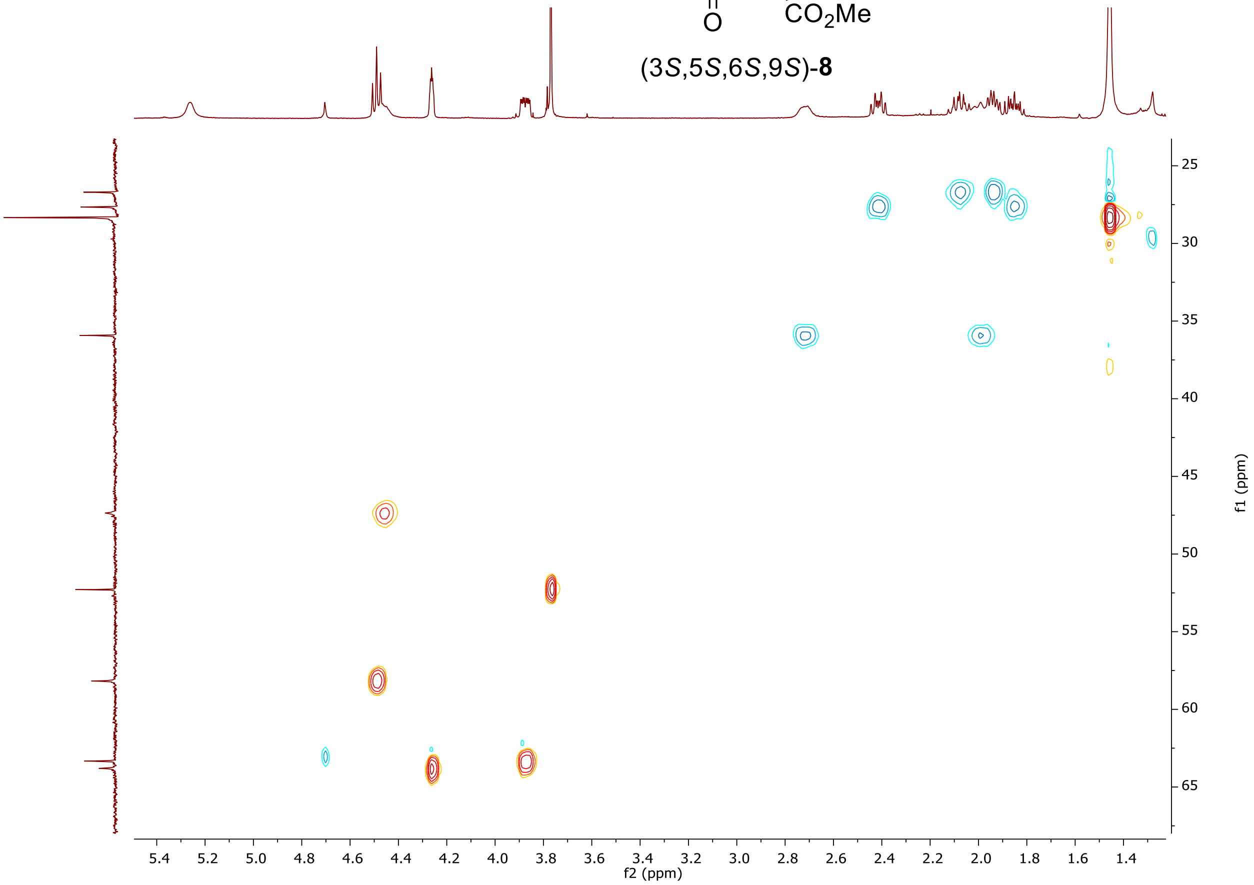

COSY, 500 MHz

Solvent: $CDCl_3$

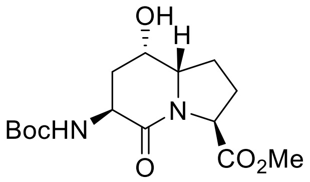

(3*S*,5*S*,6*S*,9*S*)-**8**

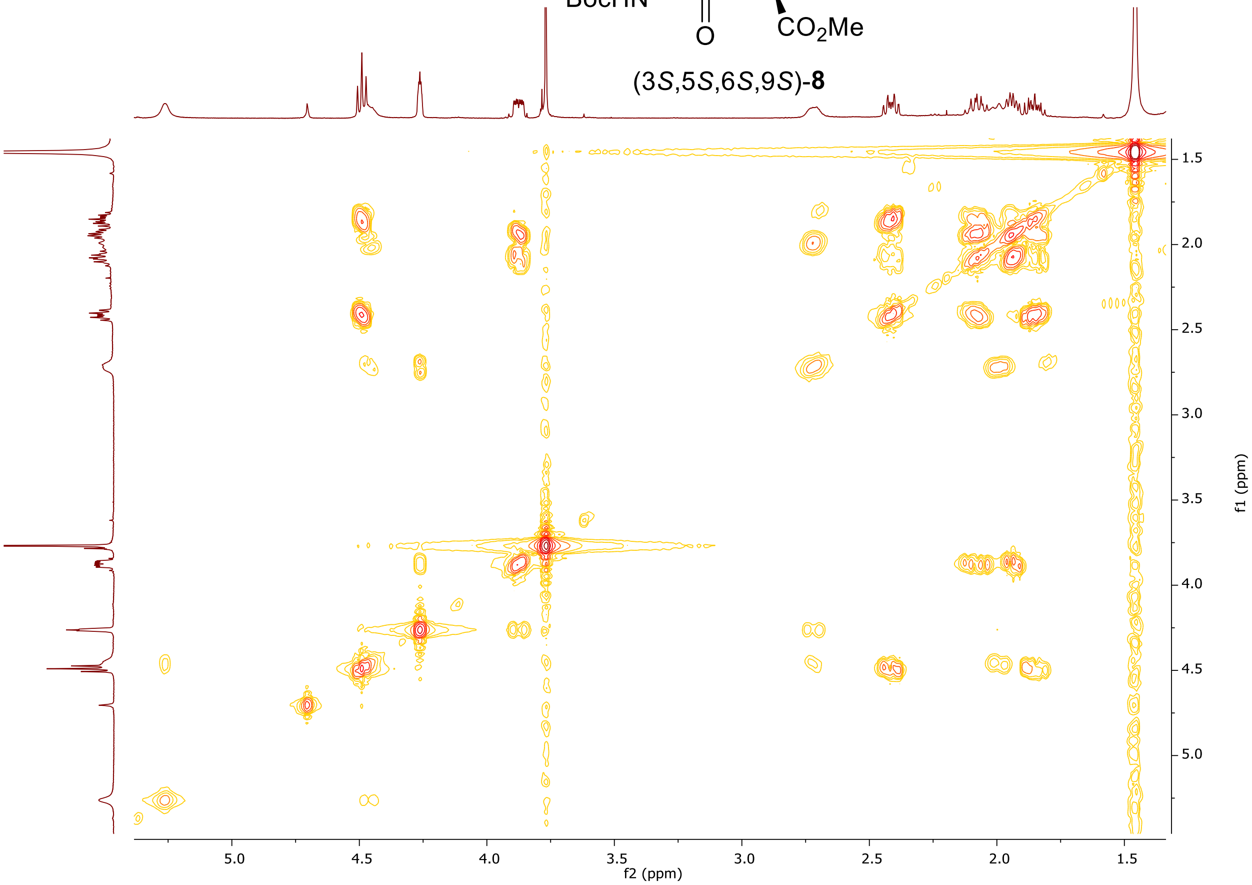

NOESY, 500 NMR

Solvent: $CDCl_3$

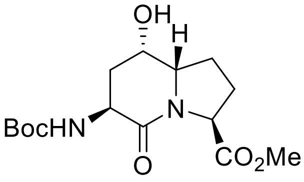

(3*S*,5*S*,6*S*,9*S*)-**8**

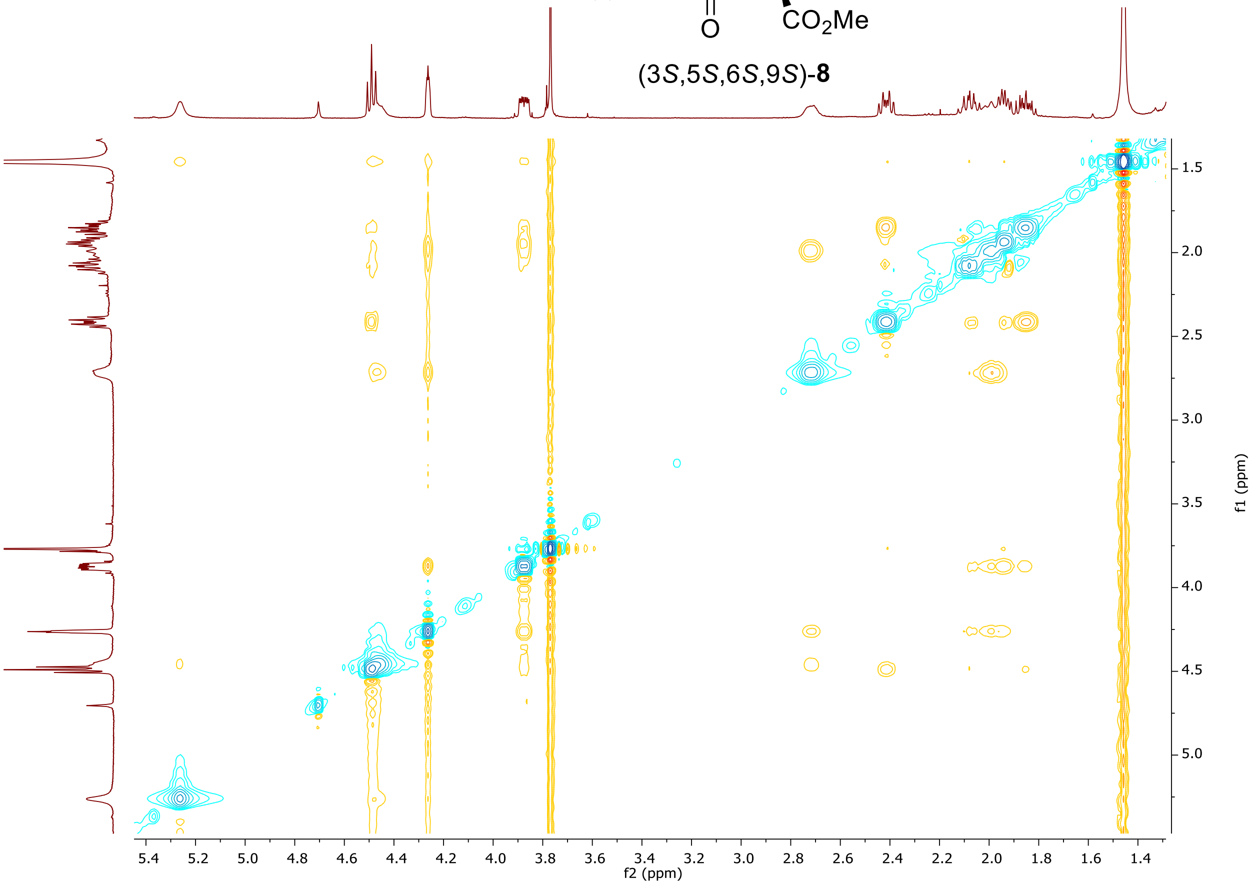

^{1}H NMR, 500 MHz

Solvent: $CDCl_3$

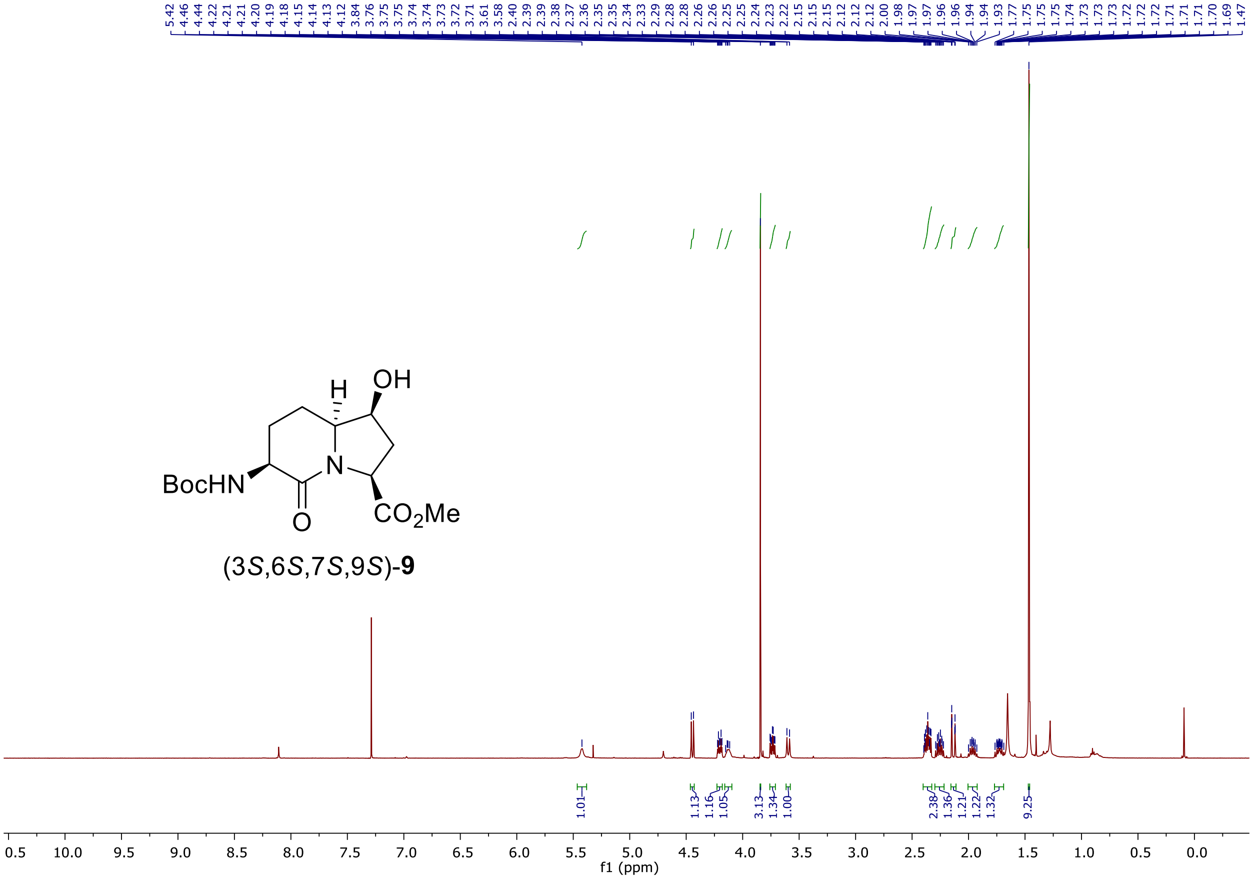

^13C NMR, 125 MHz

Solvent: CDCl$_3$

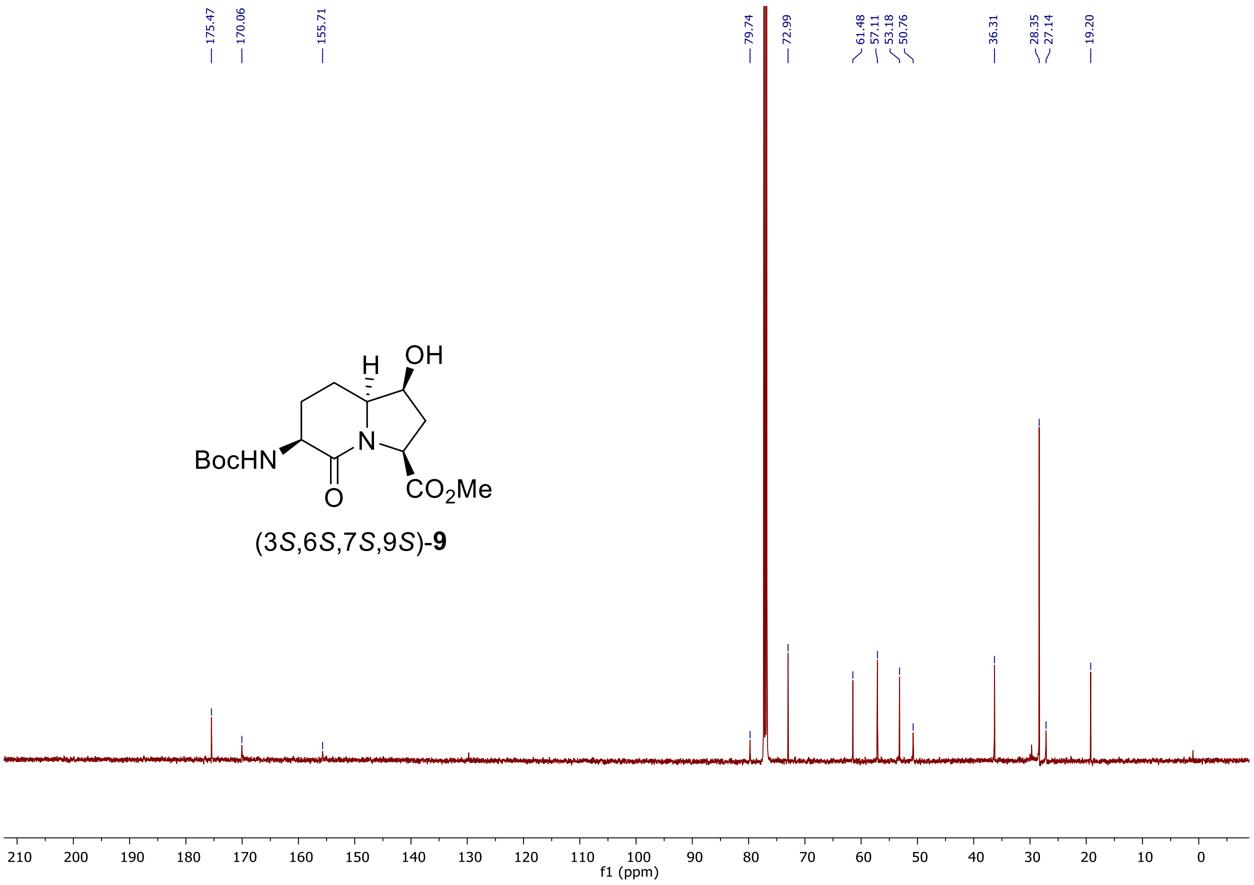

(3*S*,6*S*,7*S*,9*S*)-**9**

ed-HSQC, 500 MHz

Solvent: $CDCl_3$

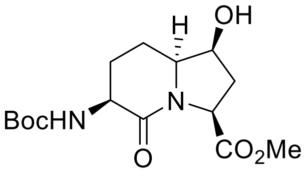

(3*S*,6*S*,7*S*,9*S*)-**9**

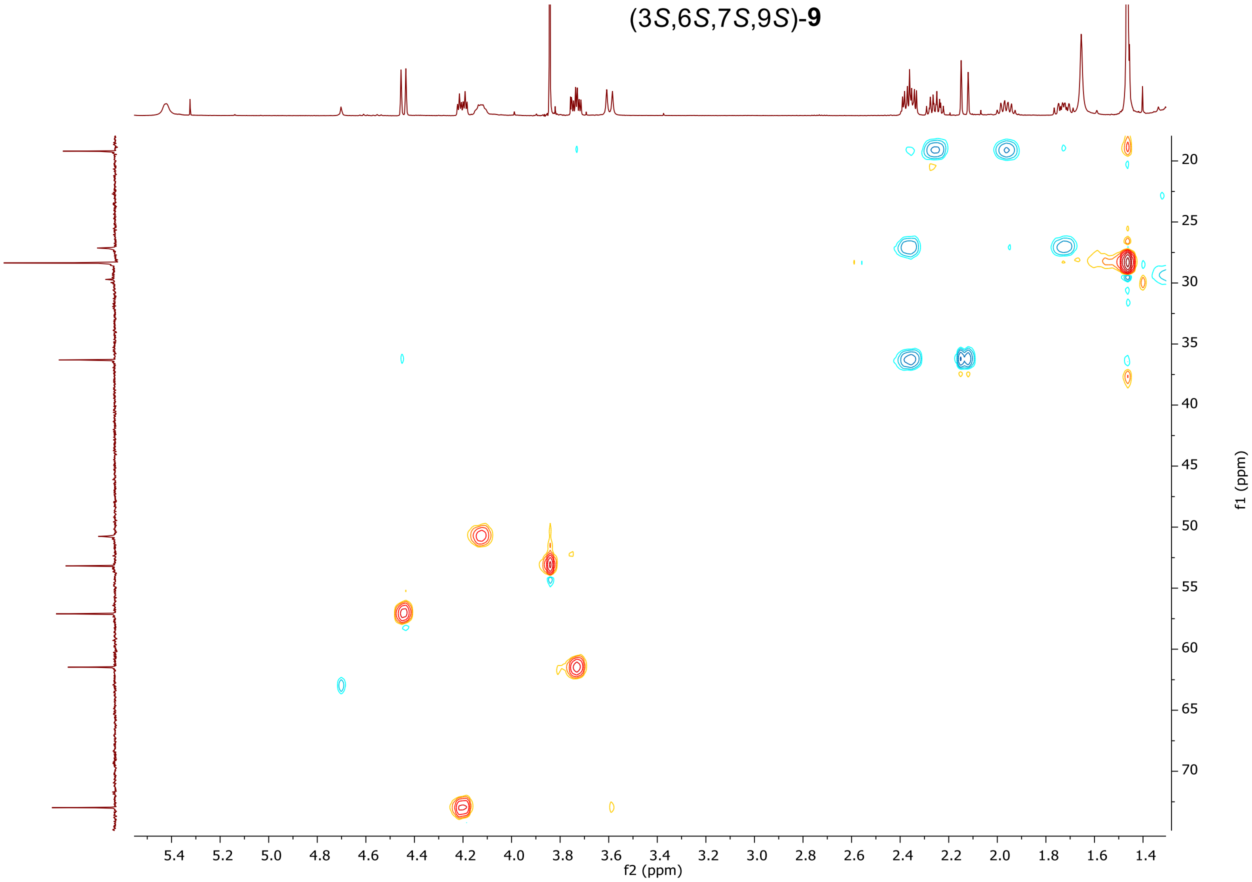

COSY, 500 MHz

Solvent: CDCl₃

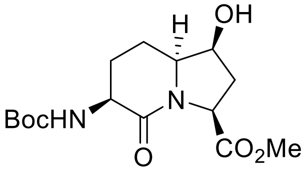

(3*S*,6*S*,7*S*,9*S*)-**9**

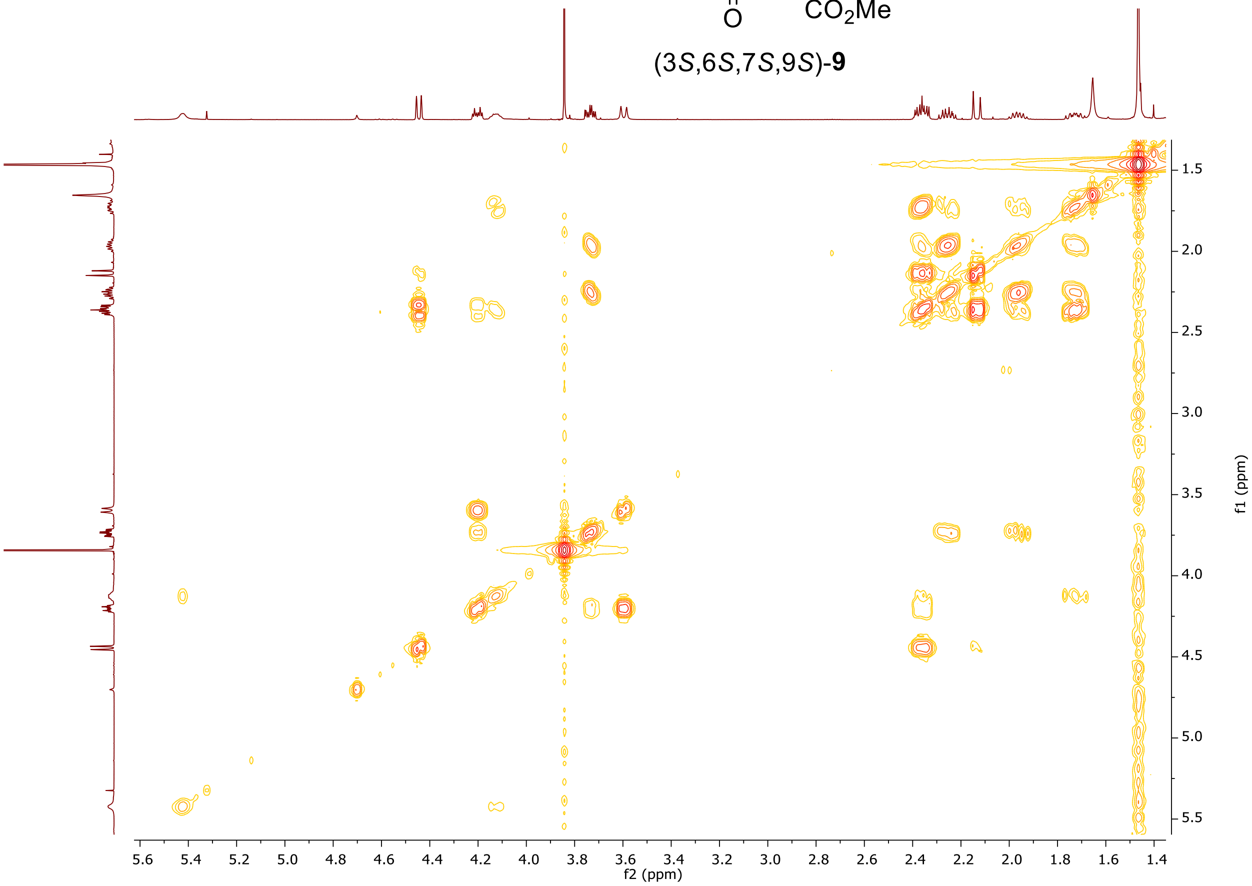

NOESY, 500 MHz

Solvent: CDCl$_3$

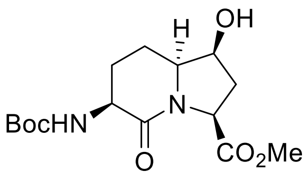

(3*S*,6*S*,7*S*,9*S*)-**9**

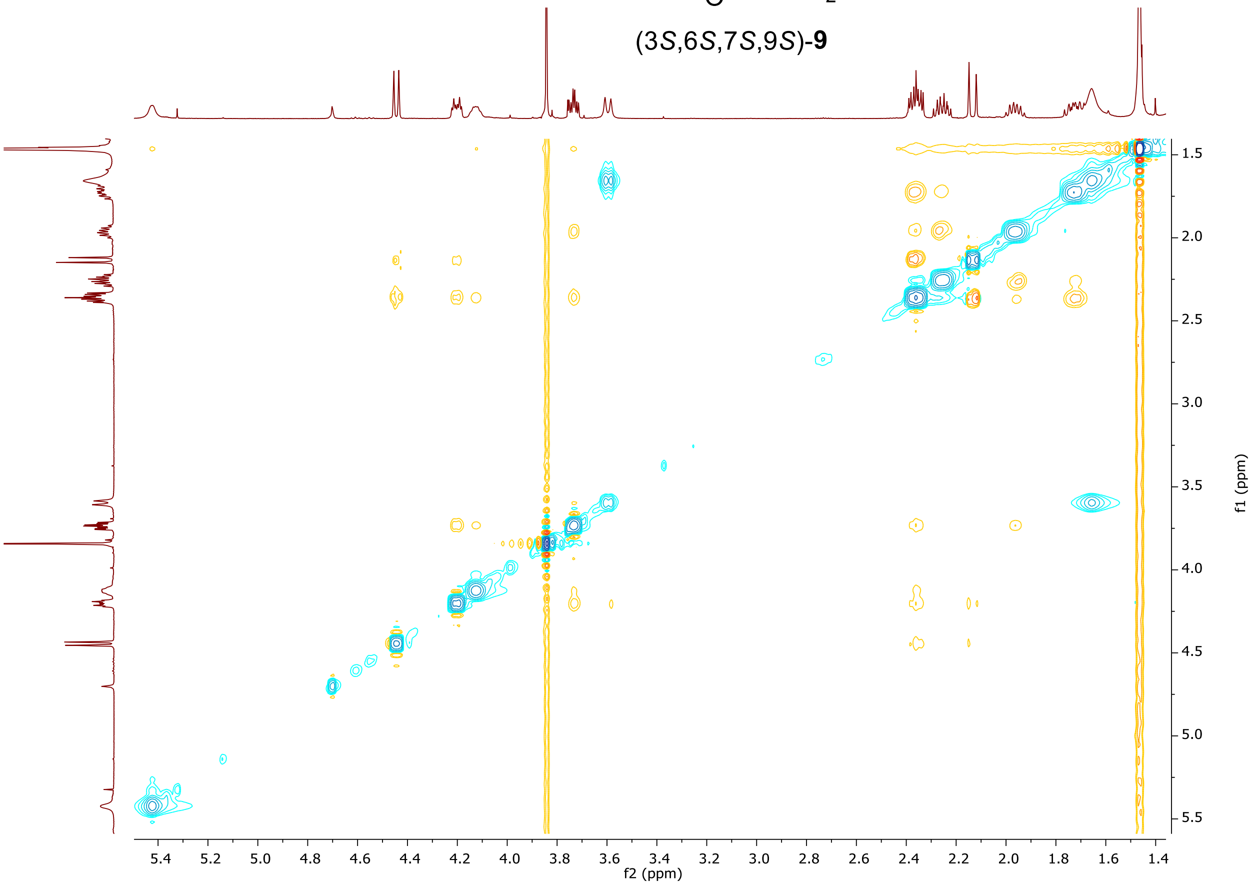

^{1}H NMR, 500 MHz

Solvent: $CDCl_3$

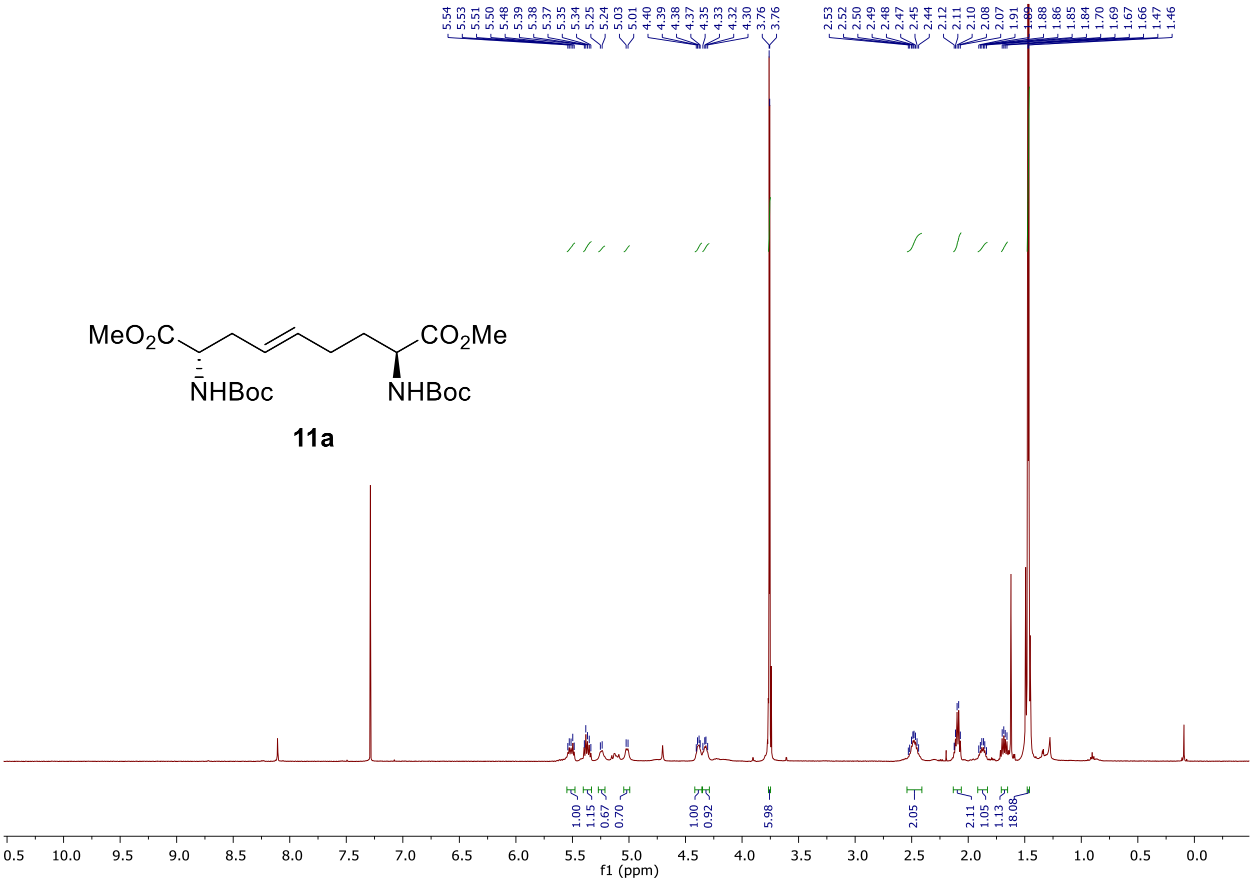

^{13}C NMR, 125 MHz

Solvent: $CDCl_3$

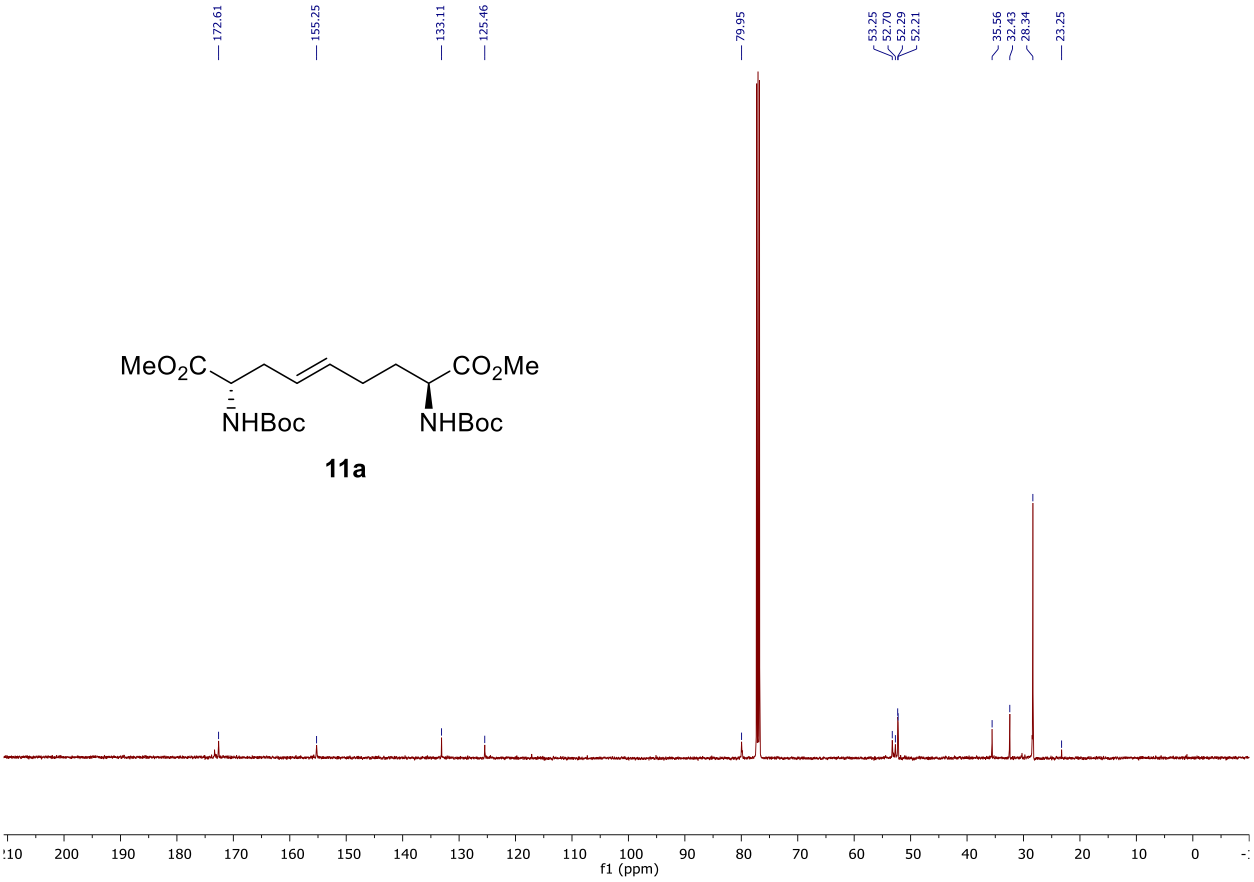

COSY, 500 MHz

Solvent: $CDCl_3$

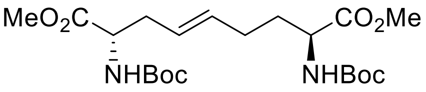

11a

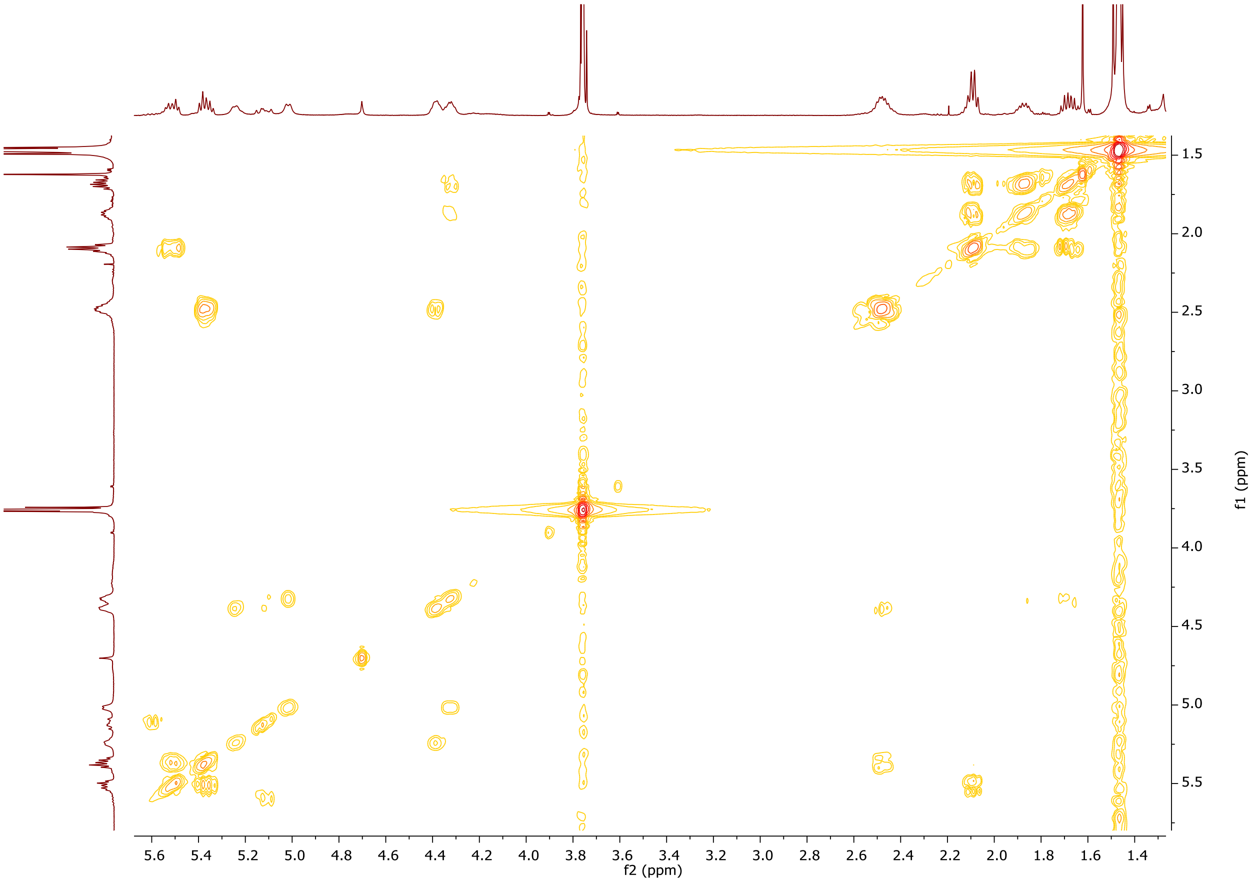

ed-HSQC, 500 MHz

Solvent: $CDCl_3$

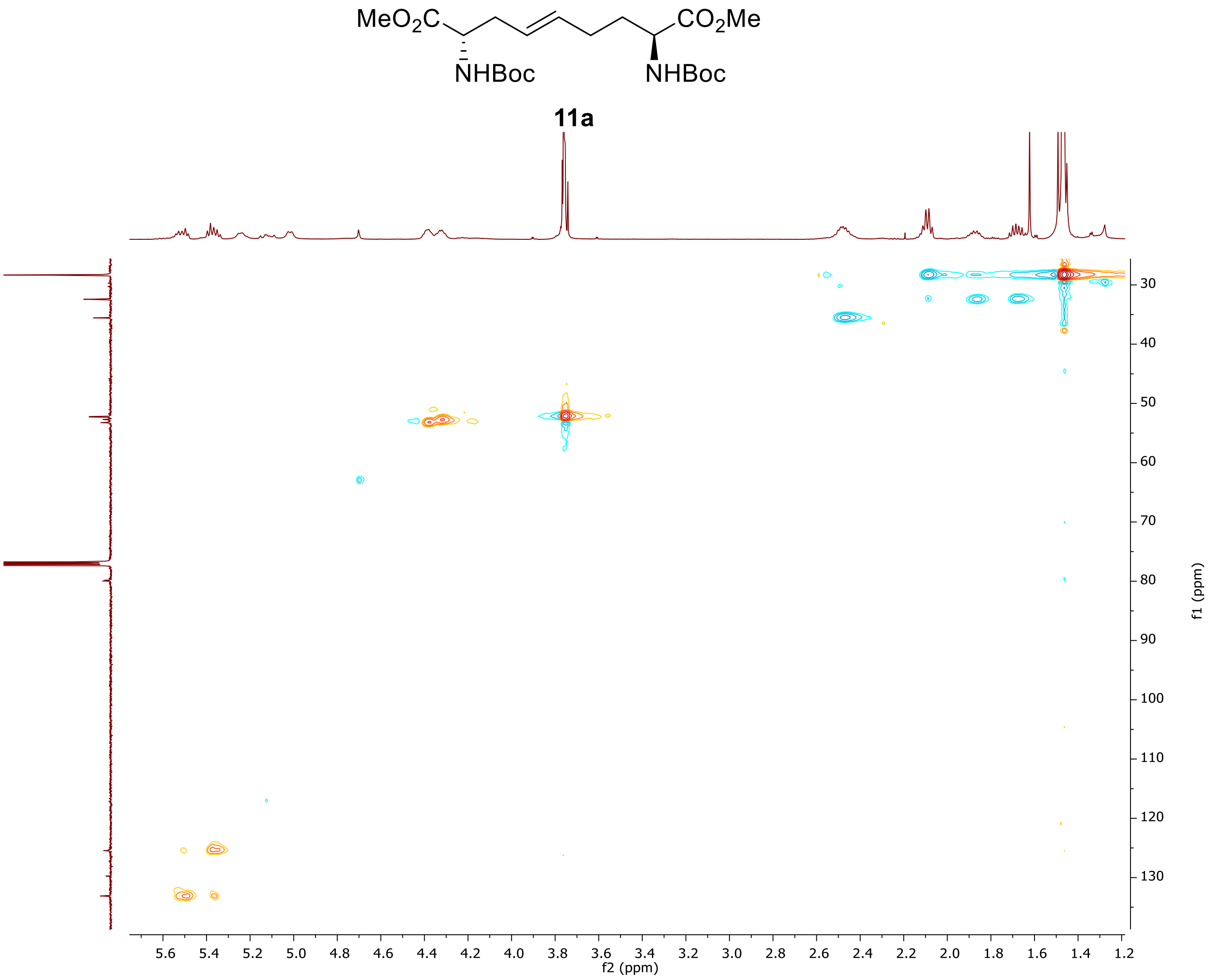

NOESY, 500 MHz

Solvent: CDCl$_3$

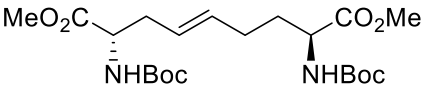

11a

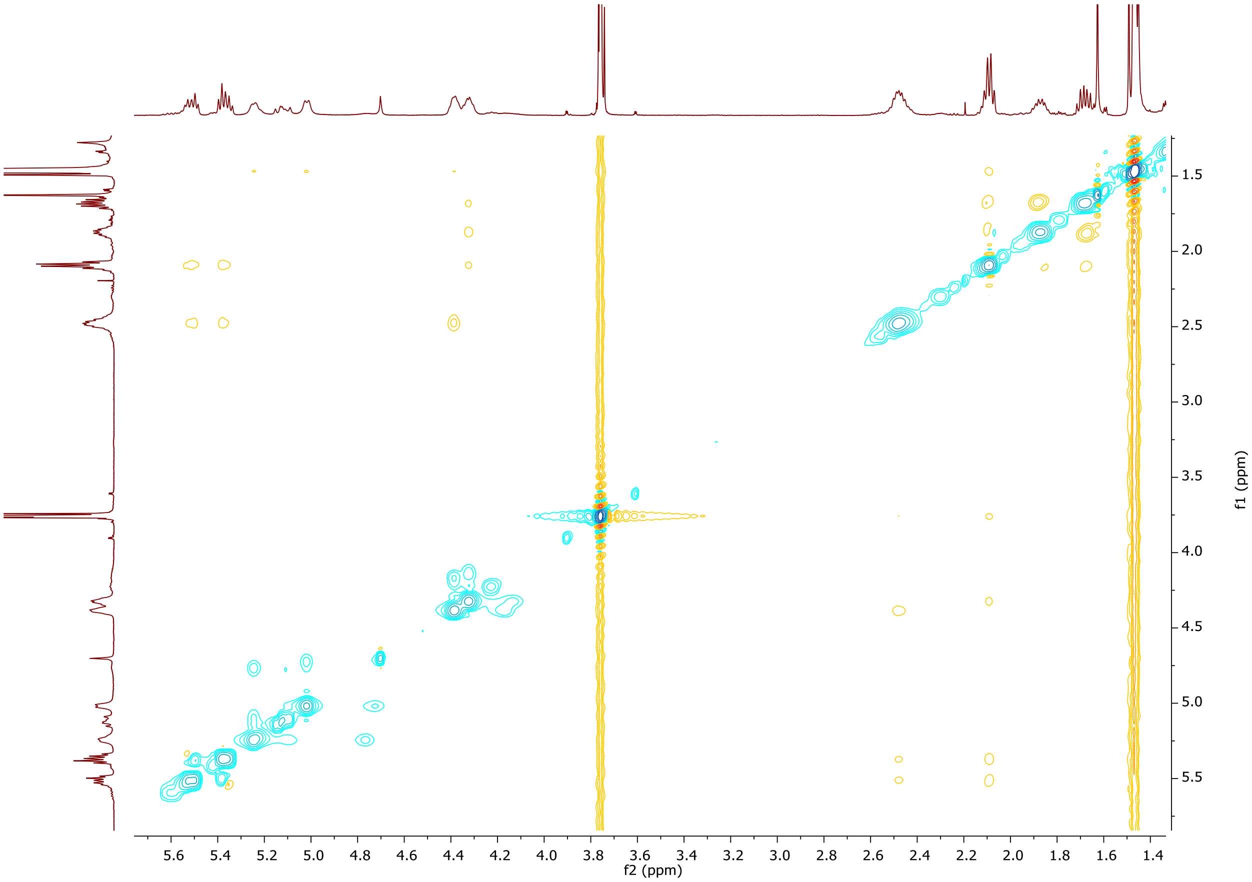

^{1}H NMR, 500 MHz

Solvent: $CDCl_3$

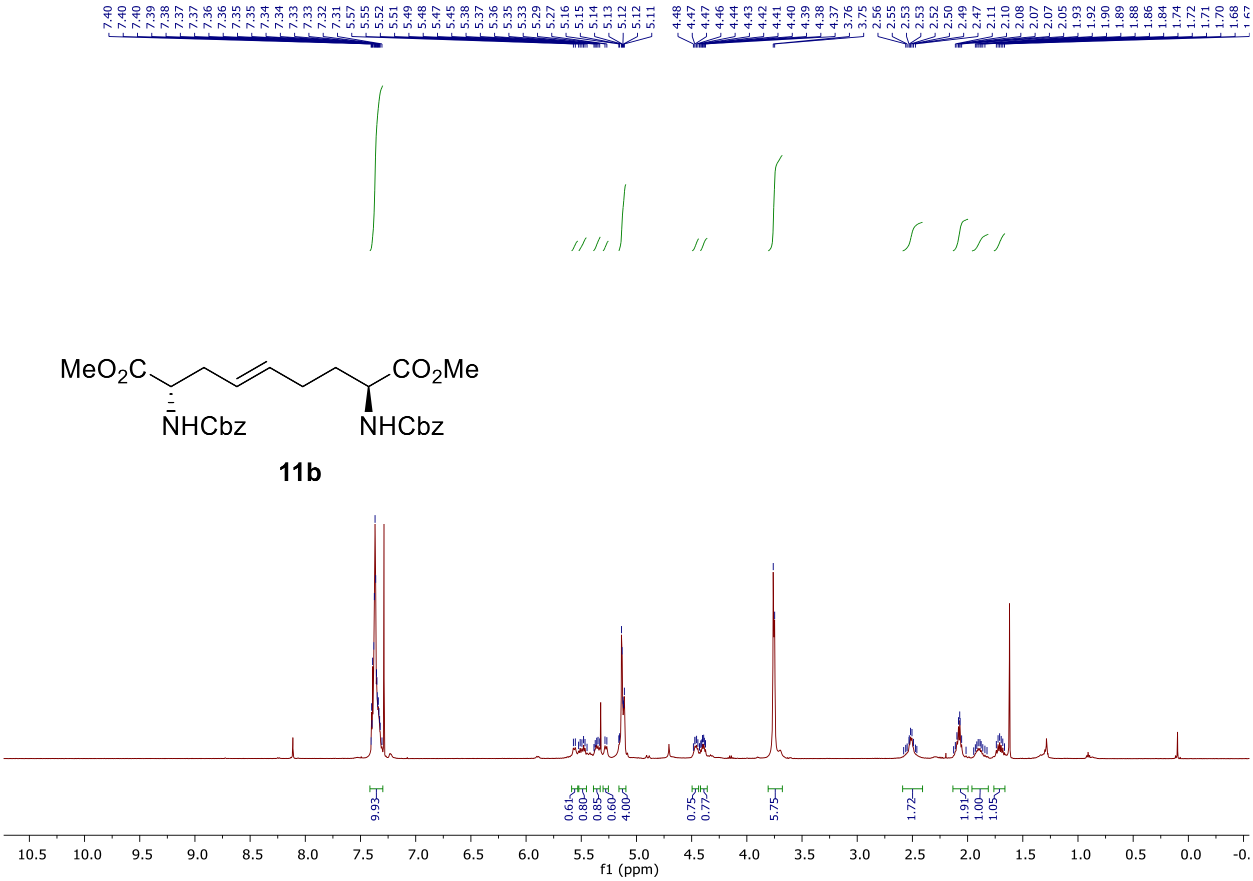

11b

^{13}C NMR, 125 MHz

Solvent: $CDCl_3$

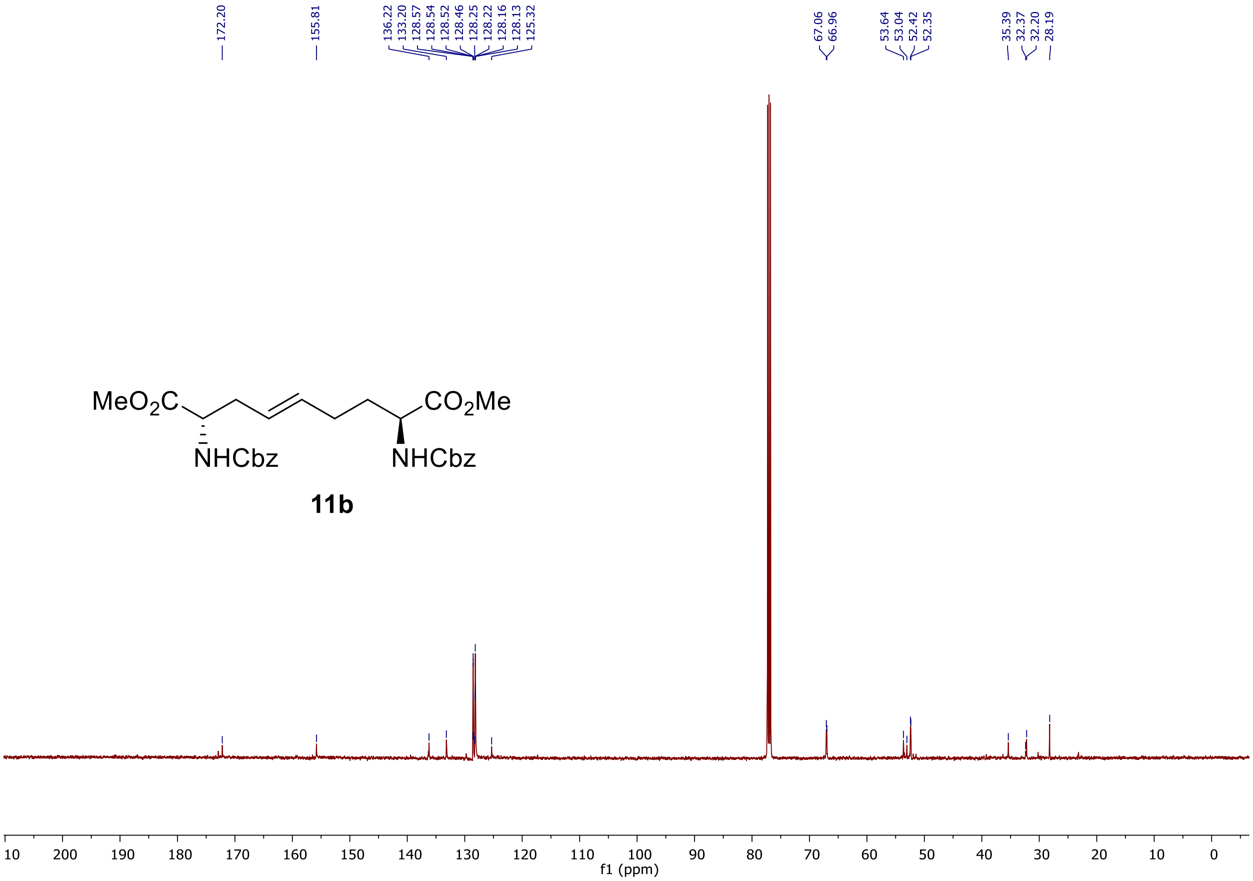

11b

COSY, 500 MHz

Solvent: $CDCl_3$

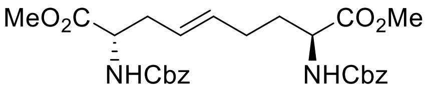

11b

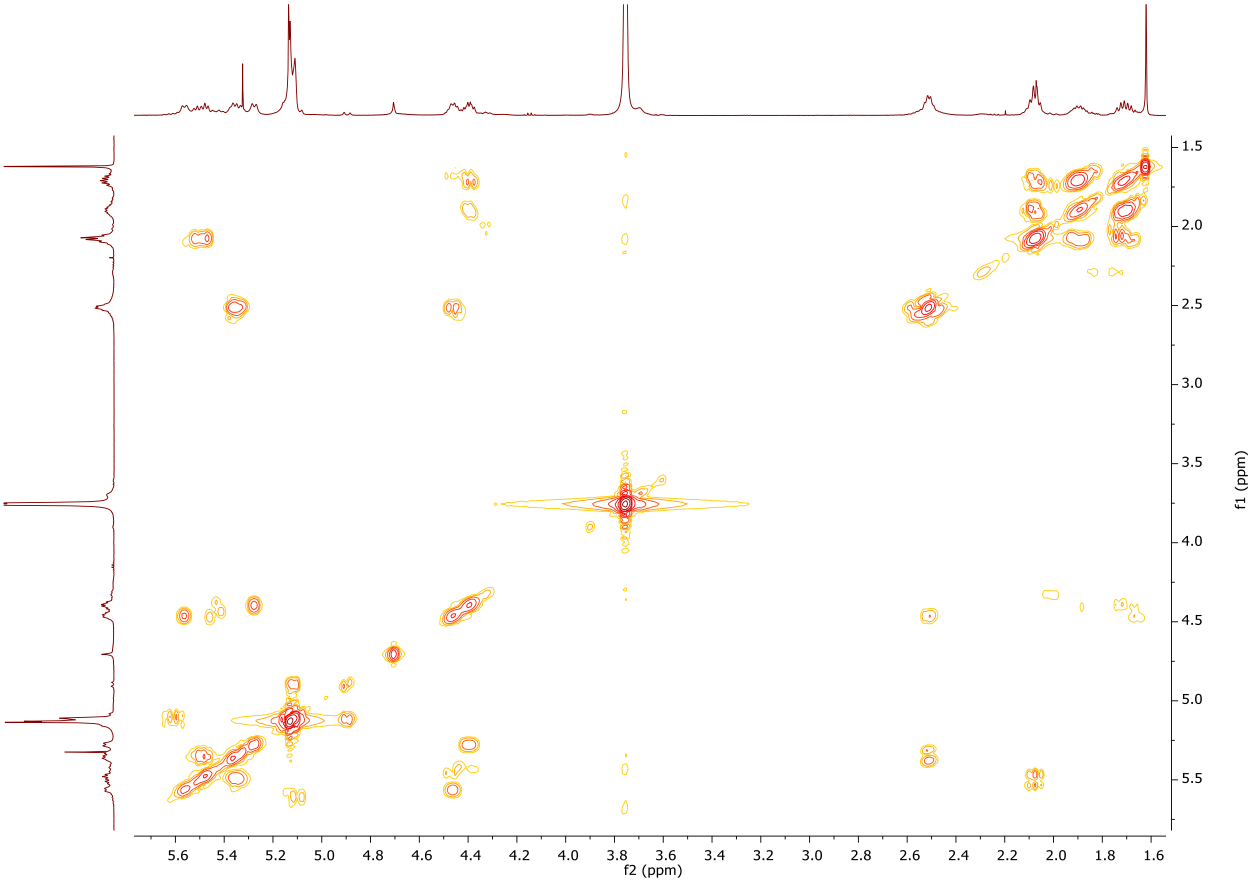

ed-HSQC, 500 MHz

Solvent: $CDCl_3$

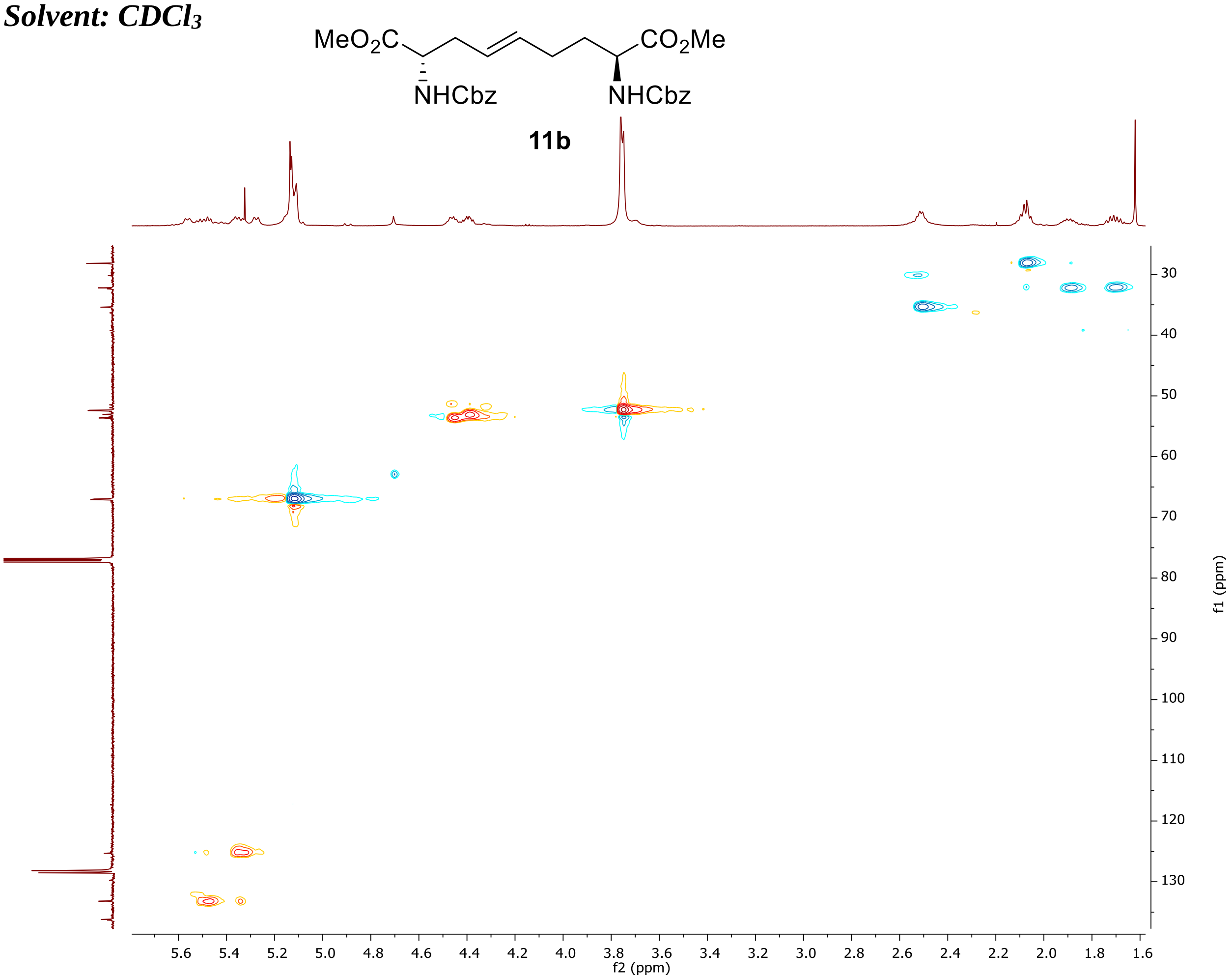

NOESY, 500 MHz

Solvent: $CDCl_3$

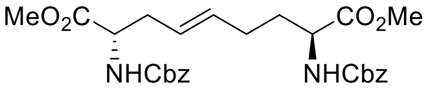

11b

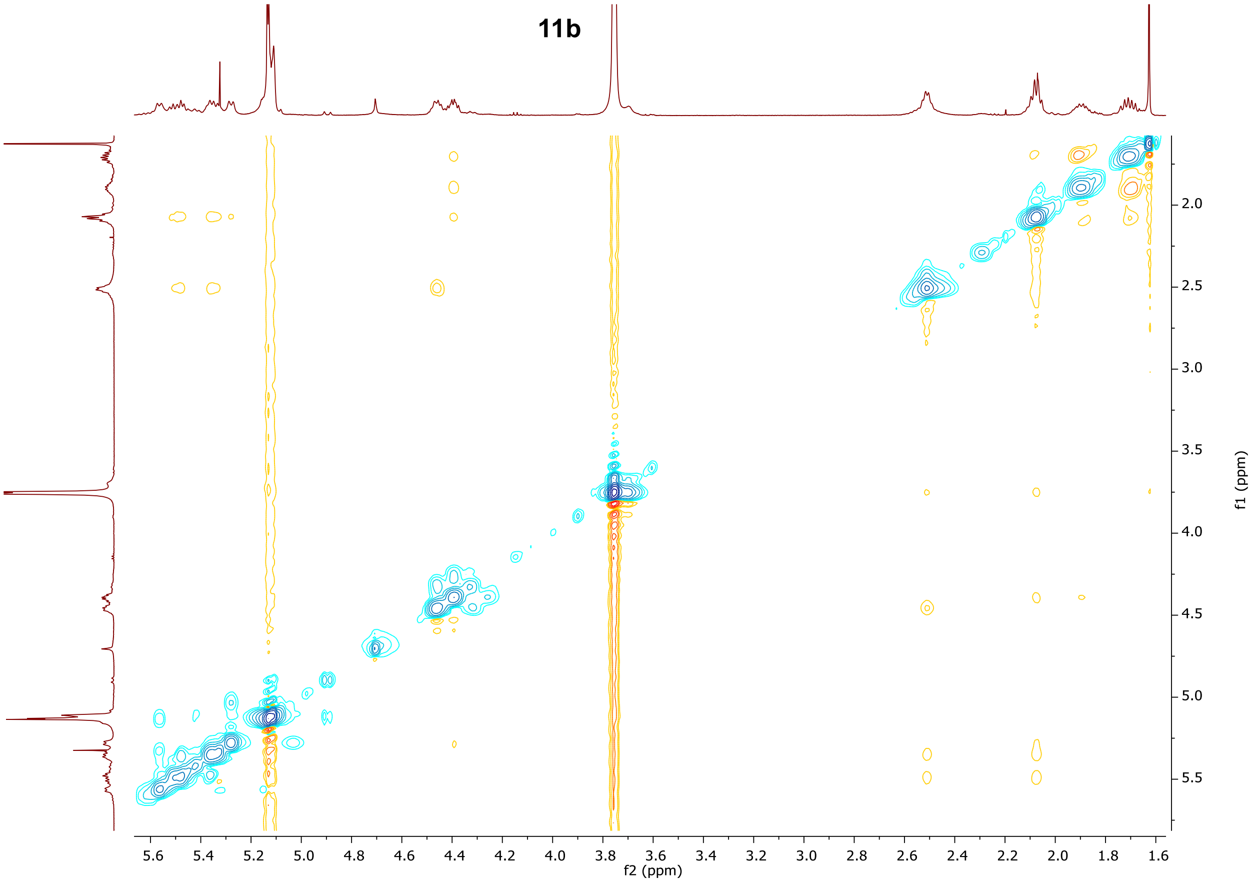

^{1}H NMR, 500 MHz,

Solvent: CD_3OD

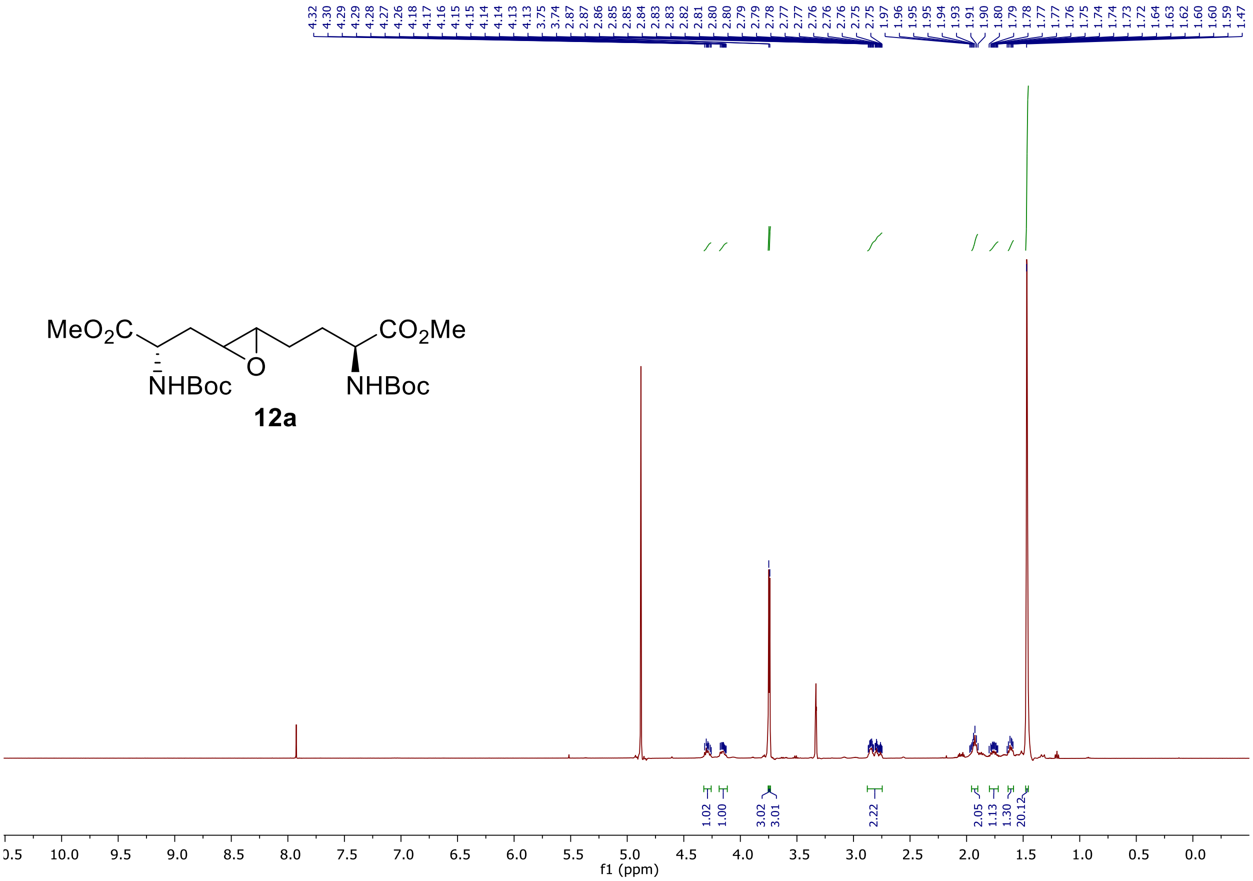

12a

13C NMR, 125 MHz

Solvent: CD_3OD

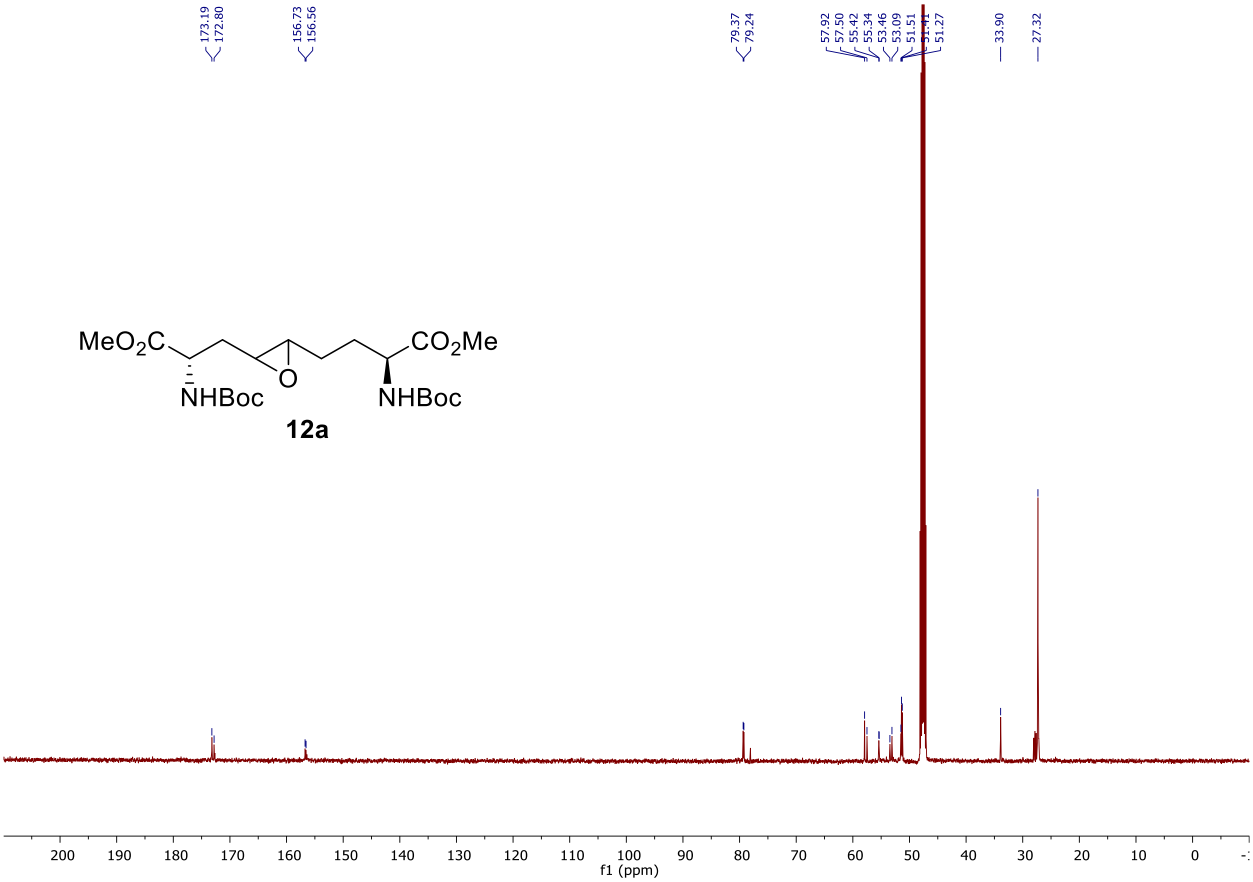

12a

^{1}H NMR, 500 MHz

Solvent: $CDCl_3$

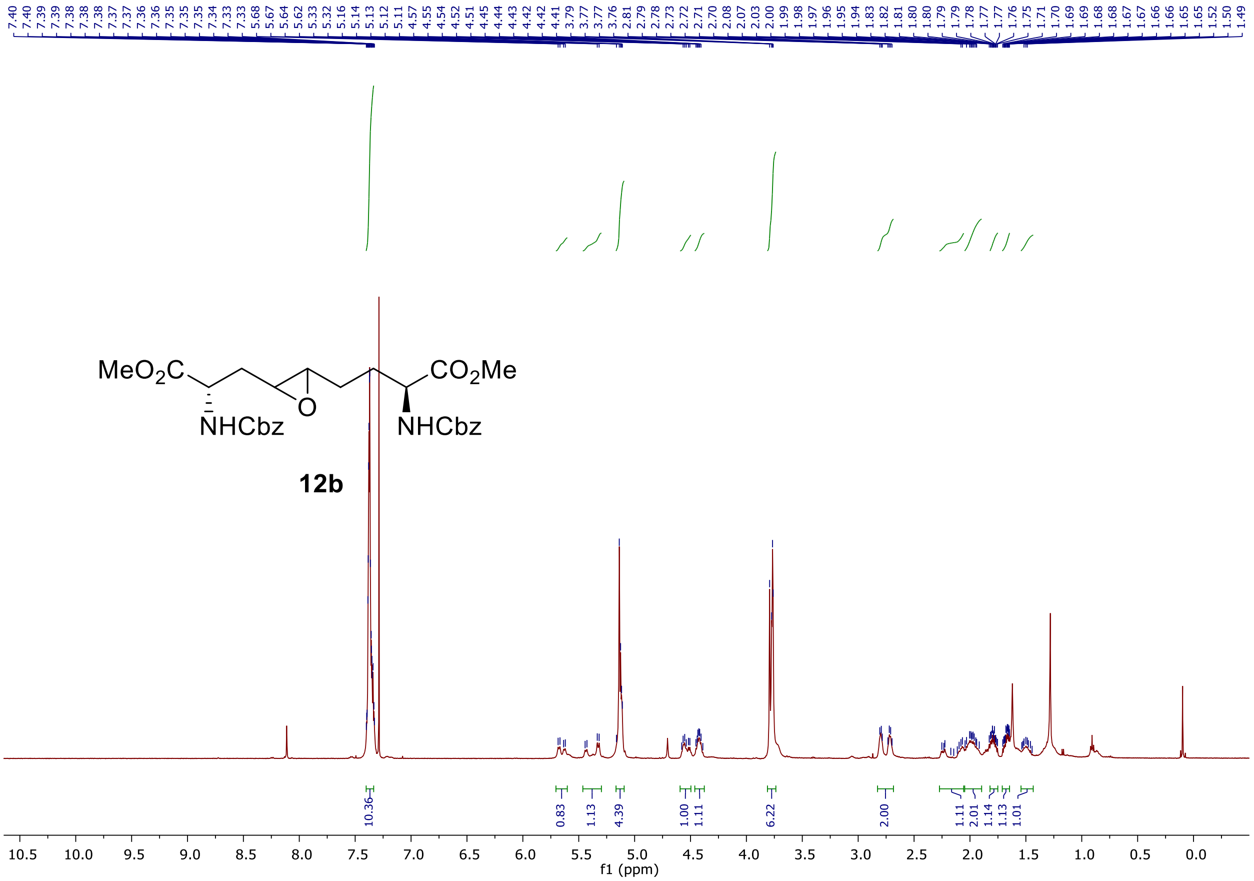

^{13}C NMR, 125 MHz

Solvent: $CDCl_3$

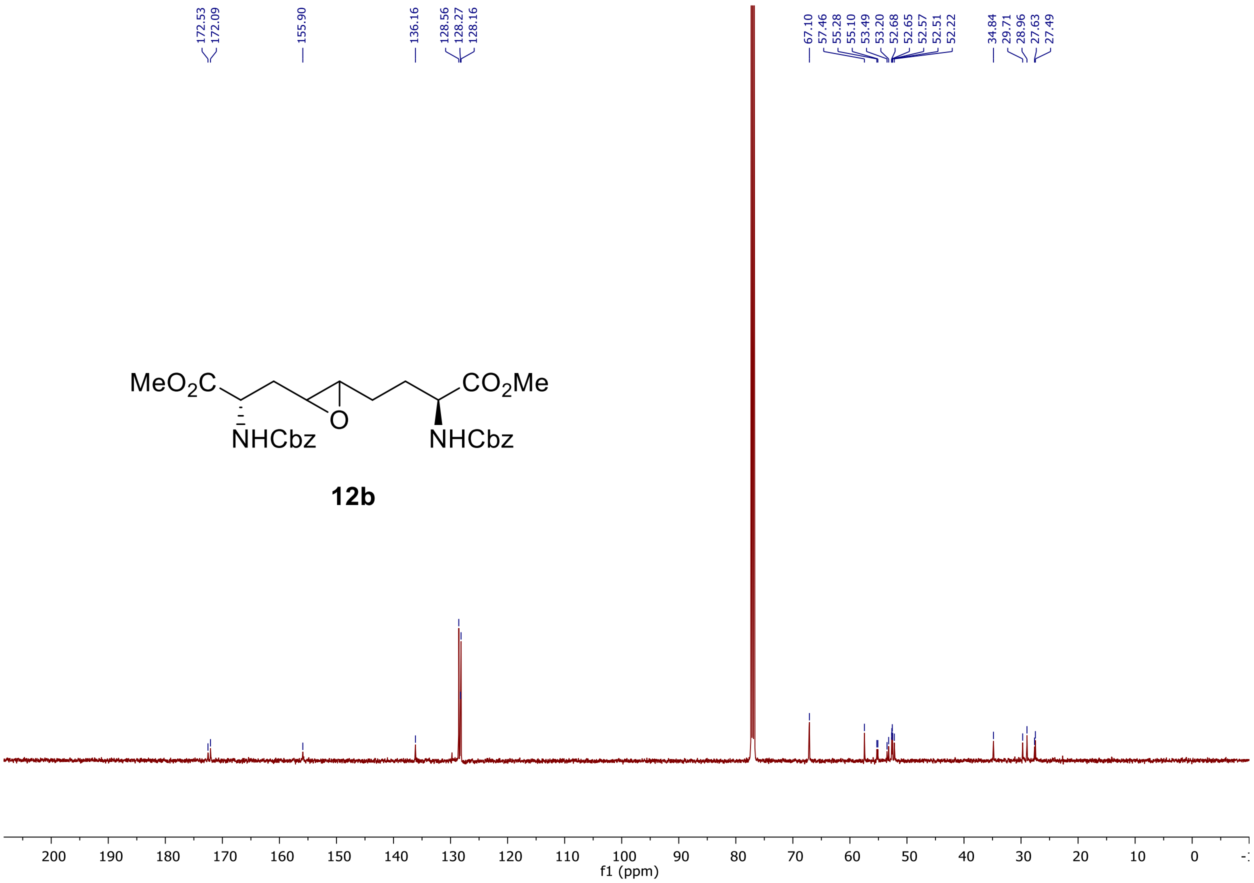

^{1}H NMR, 500 MHz

Solvent: $CDCl_3$

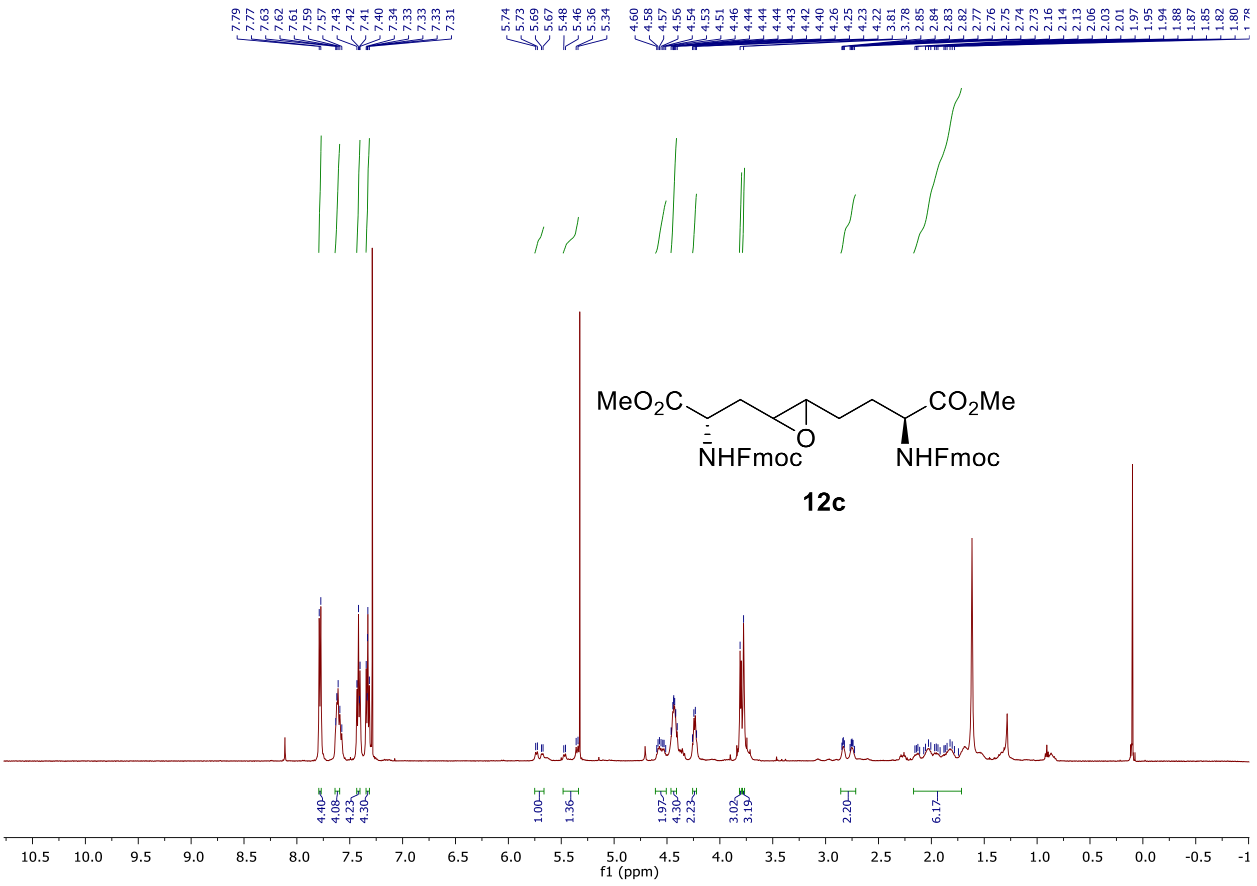

13C NMR, 125 MHz

Solvent: $CDCl_3$

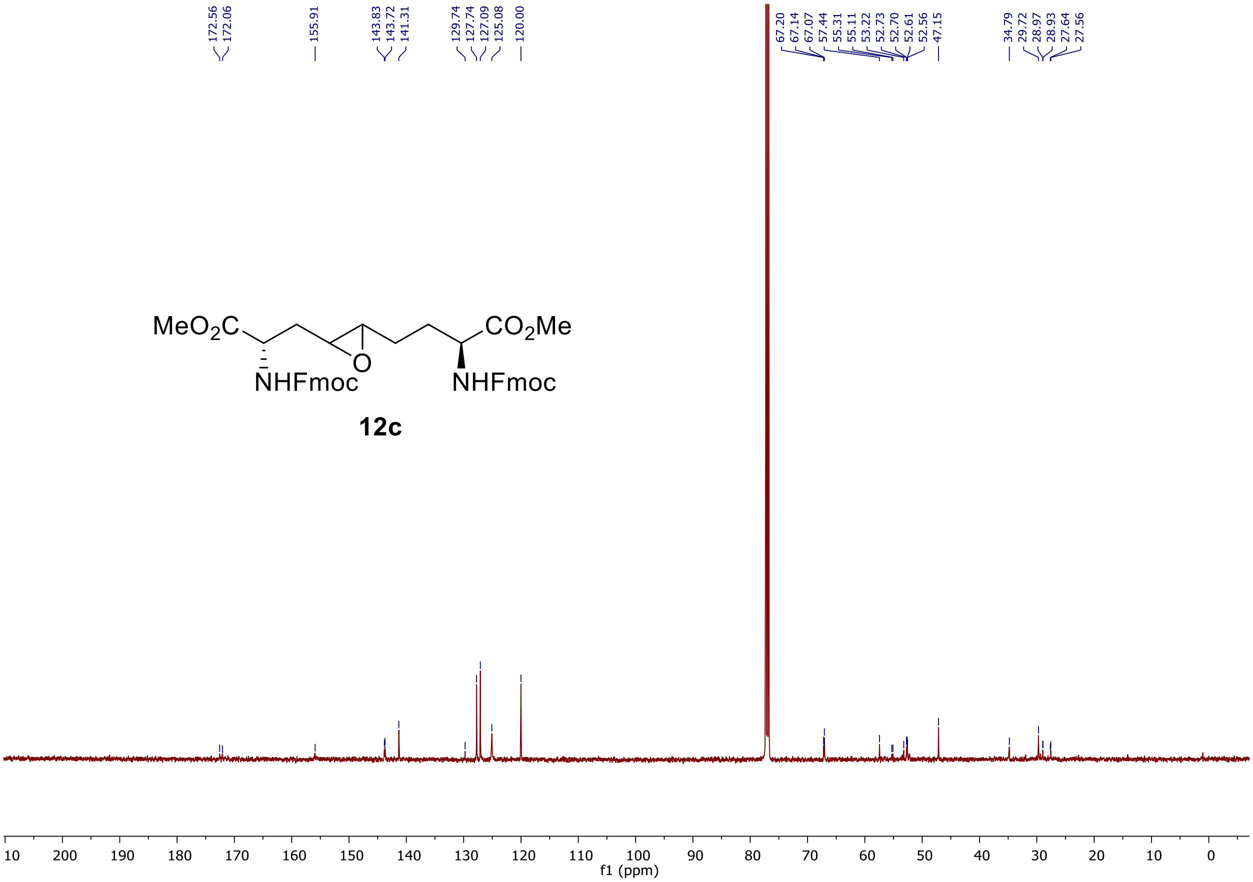

^{1}H NMR, 500 MHz

Solvent: DMSO-D$_6$

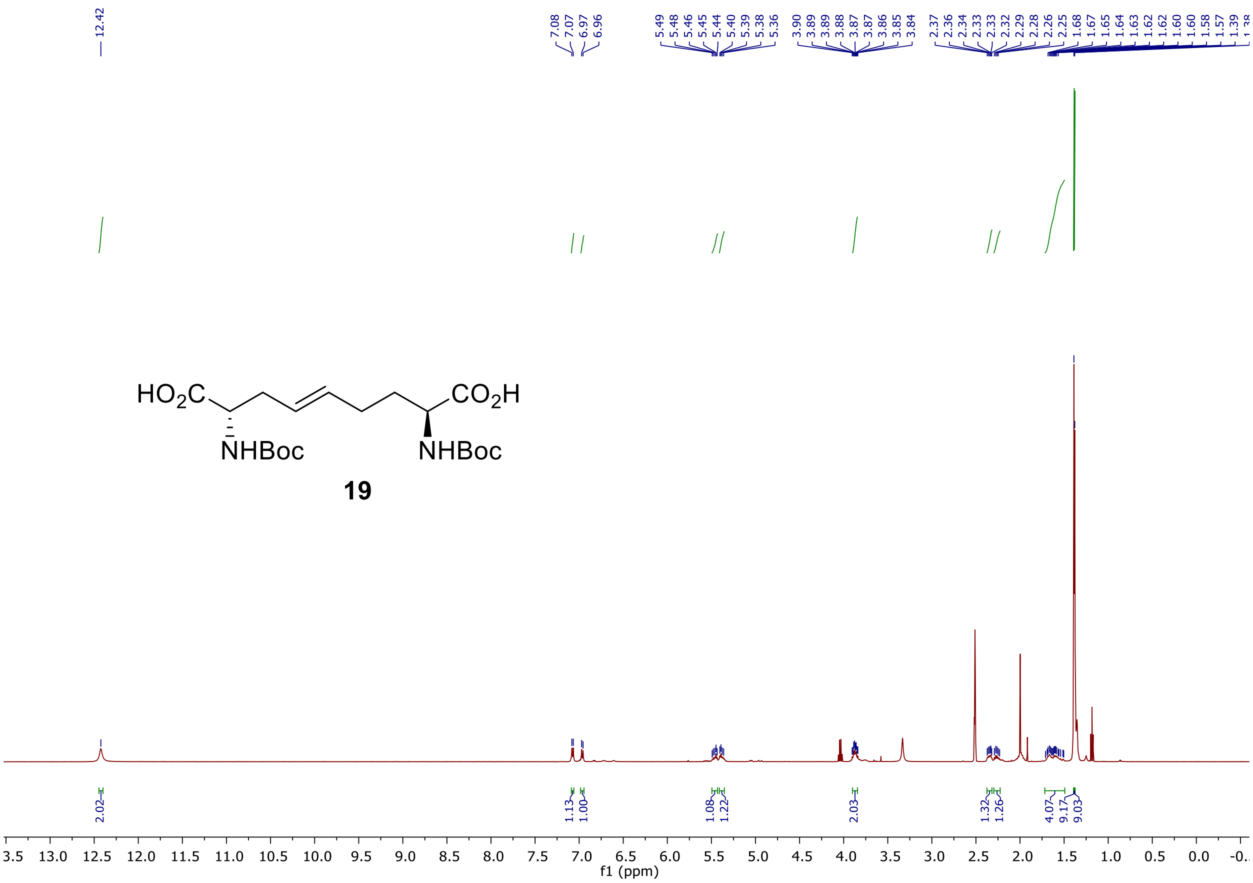

^{13}C NMR, 125 MHz

Solvent: DMSO-D$_6$

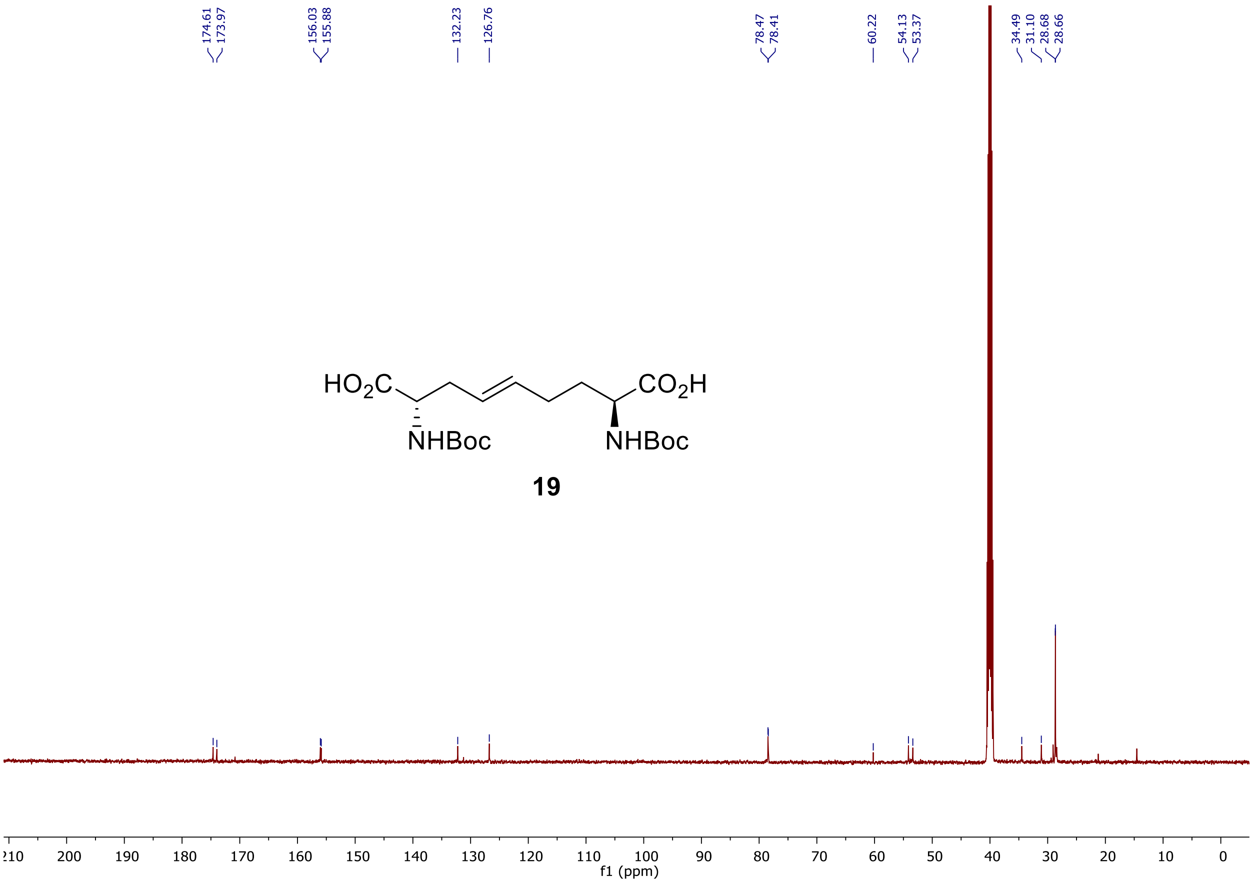

^{1}H NMR, 500 MHz

Solvent: CDCl₃

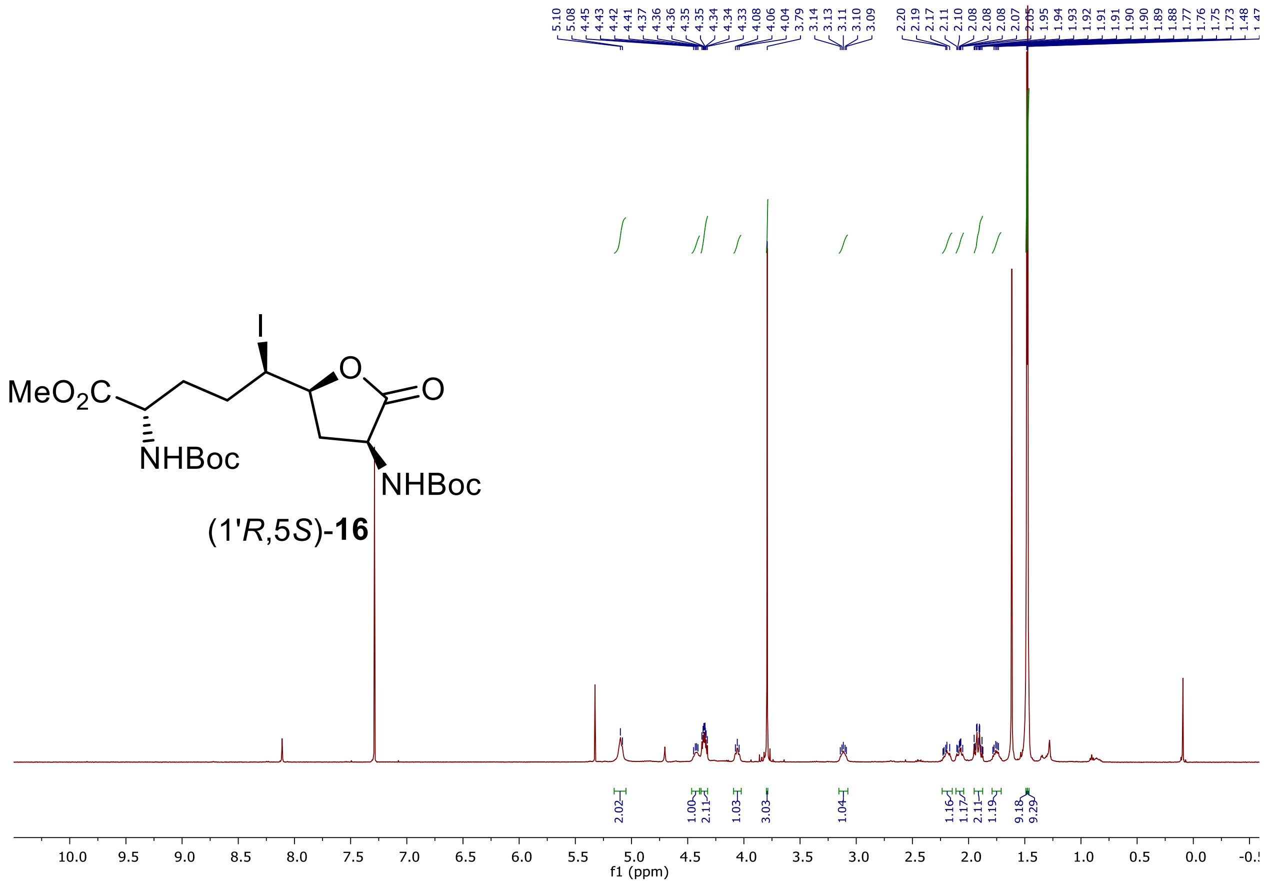

^{13}C NMR, 125 MHz

Solvent: $CDCl_3$

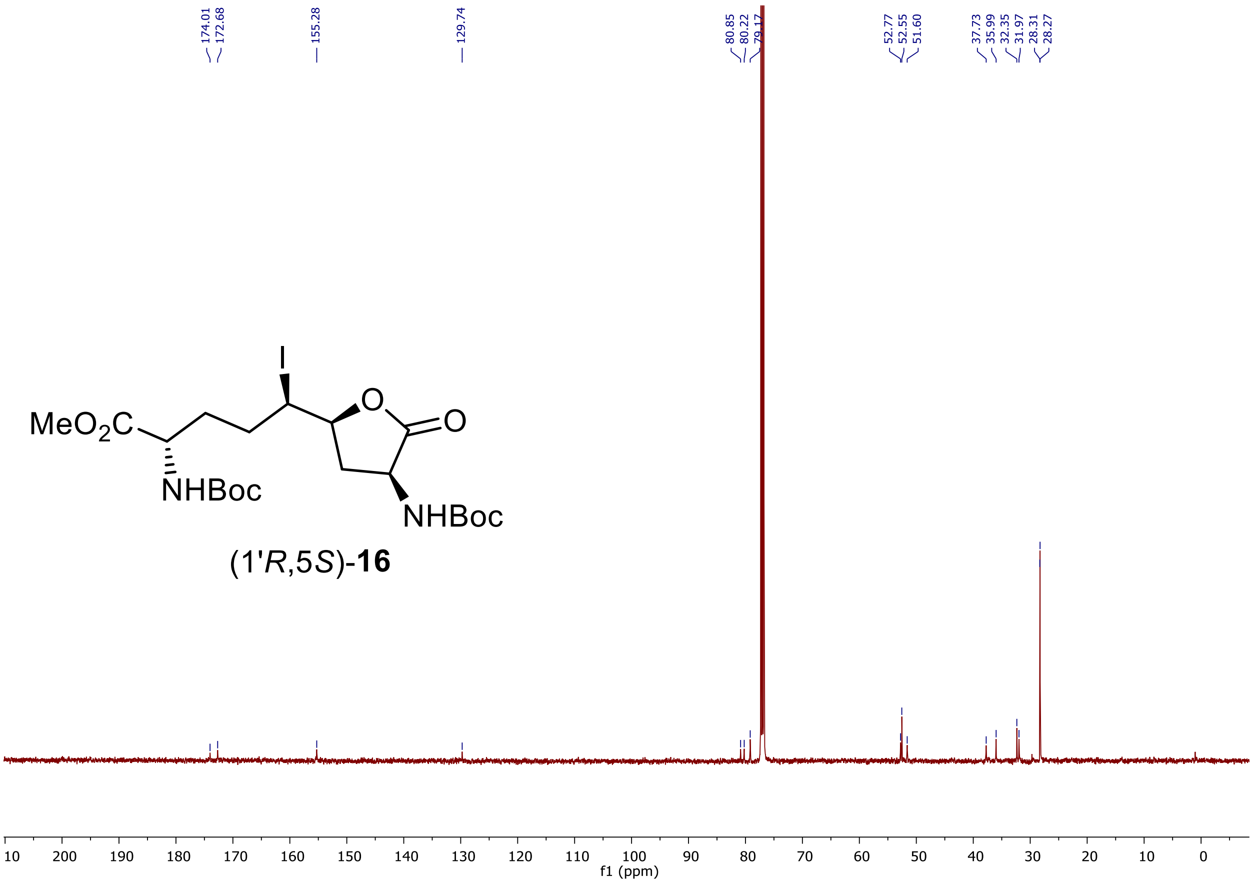

X-ray data

Methyl 3-*N*-(Boc)amino-7-hydroxy-indolizidin-2-one-9-carboxylate [(3*S*,6*S*,7*S*,9*S*)-3, LUB 1421]

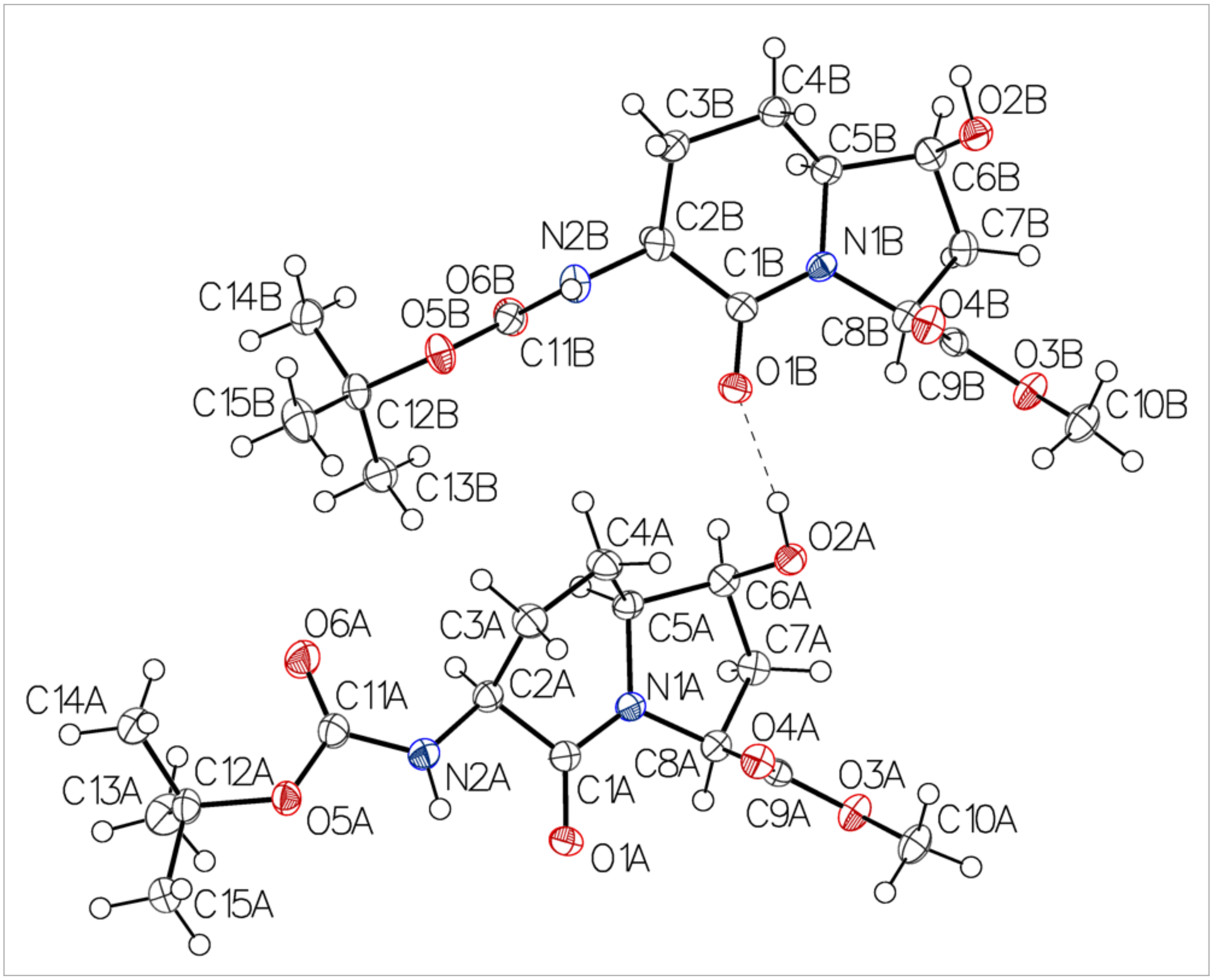

Table 1 Crystal data and structure refinement for lub1421.

Identification code	lub1421
Empirical formula	$C_{15}H_{24}N_2O_6$
Formula weight	328.36
Temperature/K	100
Crystal system	monoclinic
Space group	I2
a/Å	12.8006(6)
b/Å	5.3251(2)
c/Å	48.103(2)
α/°	90

β/°	92.926(2)
γ/°	90
Volume/Å^3	3274.6(2)
Z	8
ρ_{calc}g/cm^3	1.332
μ/mm^{-1}	0.863
F(000)	1408.0
Crystal size/mm^3	0.28 × 0.12 × 0.045
Radiation	Cu Kα (λ = 1.54178)
2Θ range for data collection/°	3.678 to 143.742
Index ranges	-15 ≤ h ≤ 15, -6 ≤ k ≤ 5, -59 ≤ l ≤ 59
Reflections collected	29804
Independent reflections	5590 [R_{int} = 0.0479, R_{sigma} = 0.0375]
Data/restraints/parameters	5590/3/436
Goodness-of-fit on F^2	1.075
Final R indexes [I>=2σ (I)]	R_1 = 0.0355, wR_2 = 0.0887
Final R indexes [all data]	R_1 = 0.0374, wR_2 = 0.0897
Largest diff. peak/hole / e Å^{-3}	0.22/-0.19
Flack parameter	0.05(19)

Table 2 Fractional Atomic Coordinates (×10^4) and Equivalent Isotropic Displacement Parameters (Å^2×10^3) for lub1421. U_{eq} is defined as 1/3 of the trace of the orthogonalised U_{IJ} tensor.

Atom	*x*	*y*	*z*	U(eq)
O1A	7550.5(13)	1894(4)	5970.0(3)	22.0(4)
O4B	1326.4(14)	10777(4)	5623.7(3)	24.2(4)
O2A	3751.2(14)	4584(4)	5634.2(3)	22.6(4)
O1B	2350.8(13)	6095(4)	6002.6(3)	22.9(4)
O6B	1992.0(14)	802(4)	6578.9(4)	24.1(4)
O4A	6277.6(13)	5971(4)	5592.1(3)	22.1(4)
O3B	1325.6(15)	8957(4)	5201.7(3)	24.6(4)
O5A	8555.1(13)	2821(4)	6877.3(3)	23.7(4)
O5B	3065.0(14)	3424(4)	6837.0(3)	24.3(4)
O3A	6095.7(14)	3849(4)	5189.3(3)	24.1(4)
O2B	-1157.5(15)	9343(4)	5610.2(4)	26.0(4)
O6A	6838.2(15)	1781(5)	6924.1(4)	37.4(5)
N1B	633.0(15)	6216(4)	5857.1(4)	18.7(4)
N1A	5775.9(15)	1862(4)	5897.2(4)	18.6(4)

Table 2 Fractional Atomic Coordinates (×10[4]) and Equivalent Isotropic Displacement Parameters (Å[2]×10[3]) for lub1421. U_{eq} is defined as 1/3 of the trace of the orthogonalised U_{IJ} tensor.

Atom	*x*	*y*	*z*	U(eq)
N2B	1860.9(17)	5063(5)	6546.2(4)	22.3(4)
N2A	7440.2(16)	3038(5)	6511.5(4)	24.2(5)
C11B	2285.9(19)	2897(5)	6646.3(5)	19.9(5)
C9B	1163.4(19)	8989(5)	5476.9(5)	18.5(5)
C1A	6657.0(18)	2076(5)	6055.8(5)	18.8(5)
C9A	6063.9(18)	4071(5)	5465.9(5)	18.6(5)
C1B	1418.0(18)	5889(5)	6049.4(5)	18.2(5)
C8A	5756.1(18)	1600(5)	5596.7(5)	19.2(5)
C4B	-658.8(19)	7494(5)	6179.2(5)	22.4(5)
C11A	7547(2)	2444(6)	6785.0(5)	25.3(6)
C12A	8963(2)	1548(5)	7131.2(5)	23.4(5)
C5A	4728.5(18)	2186(5)	6007.3(5)	19.4(5)
C4A	4705(2)	4554(6)	6187.4(5)	24.5(6)
C7A	4623.4(18)	776(5)	5528.7(5)	21.1(5)
C6A	3990.2(19)	2151(5)	5741.2(5)	20.8(5)
C2B	1029.4(19)	5097(5)	6331.4(5)	20.5(5)
C12B	3621(2)	1382(5)	6990.9(5)	23.0(5)
C7B	-311.2(19)	5755(5)	5432.9(5)	21.4(5)
C6B	-1054.4(19)	6698(5)	5647.9(5)	22.5(5)
C2A	6453.0(18)	2637(5)	6358.7(5)	20.9(5)
C8B	783.7(19)	6421(5)	5560.1(5)	18.9(5)
C5B	-473.2(18)	5945(5)	5921.7(5)	19.9(5)
C3A	5719(2)	4906(6)	6371.2(5)	25.1(5)
C3B	98.1(19)	6663(5)	6424.7(5)	21.7(5)
C10A	6431(2)	6085(6)	5048.9(5)	27.7(6)
C14A	8467(2)	2529(6)	7390.1(5)	27.7(6)
C14B	2863(2)	-77(6)	7162.8(5)	29.5(6)
C10B	1712(2)	11258(5)	5087.1(5)	27.1(6)
C15A	10116(2)	2205(6)	7135.9(5)	30.6(6)
C15B	4395(2)	2822(6)	7182.4(6)	33.9(7)
C13B	4199(2)	-272(6)	6793.8(5)	25.7(6)
C13A	8798(3)	-1248(6)	7099.8(6)	34.2(7)

Table 3 Anisotropic Displacement Parameters (Å^2×10^3) for lub1421. The Anisotropic displacement factor exponent takes the form: -2π^2[h^{2}a*^{2}U$_{11}$+2hka*b*U$_{12}$+…].

Atom	U_{11}	U_{22}	U_{33}	U_{23}	U_{13}	U_{12}
O1A	18.2(8)	21.1(10)	26.7(8)	1.1(7)	2.7(6)	1.3(7)
O4B	33.4(10)	16.9(10)	22.4(8)	-0.5(7)	1.9(7)	-2.0(8)
O2A	26.0(9)	19.9(10)	22.3(8)	2.6(7)	5.0(7)	4.5(7)
O1B	19.1(8)	25.5(11)	24.2(8)	-1.2(7)	1.5(6)	2.0(7)
O6B	27.7(9)	16.8(10)	27.3(8)	-0.4(7)	-2.8(7)	-1.4(7)
O4A	26.2(9)	15.5(9)	24.5(8)	-1.0(7)	1.3(6)	-1.2(7)
O3B	35.6(10)	19.3(10)	19.5(8)	0.2(7)	7.4(7)	-2.9(8)
O5A	22.0(9)	26.9(11)	21.9(8)	6.4(8)	-1.3(6)	-3.4(8)
O5B	30.4(10)	16.8(10)	24.9(8)	0.8(7)	-7.4(7)	1.9(8)
O3A	32.8(10)	20.4(10)	19.5(8)	0.0(7)	4.8(7)	-3.1(8)
O2B	29.5(10)	22.8(11)	26.0(9)	5.3(8)	6.2(7)	5.2(8)
O6A	26.8(10)	57.0(15)	28.2(9)	10.1(10)	0.5(7)	-9.5(10)
N1B	20.9(10)	16.8(11)	18.9(9)	0.9(8)	4.1(7)	-1.2(8)
N1A	20.0(10)	16.3(11)	19.8(9)	1.4(8)	3.5(7)	1.4(8)
N2B	28.3(11)	16.2(12)	21.8(10)	-0.2(8)	-3.6(8)	-2.2(9)
N2A	20.5(10)	30.0(13)	22.3(10)	3.9(9)	2.0(8)	-2.2(9)
C11B	22.7(12)	19.8(14)	17.4(10)	-0.2(10)	1.9(8)	-0.1(10)
C9B	18.5(11)	17.7(13)	19.1(10)	-0.5(10)	-0.2(8)	3.8(9)
C1A	21.2(12)	12.4(12)	22.9(11)	2.2(9)	3.2(9)	0.1(9)
C9A	17.3(11)	17.1(13)	21.4(11)	-0.4(10)	1.1(8)	2.4(9)
C1B	21.2(12)	12.3(12)	21.2(10)	-2.7(9)	1.5(8)	2.0(9)
C8A	22.4(12)	15.1(13)	20.1(11)	0.0(9)	2.3(9)	-0.1(9)
C4B	22.2(12)	21.2(15)	24.1(11)	4.0(10)	4.5(9)	1.9(10)
C11A	25.3(13)	26.7(16)	23.6(11)	5.3(10)	1.0(9)	-0.8(11)
C12A	27.4(13)	22.9(15)	19.9(11)	3.8(10)	-0.1(9)	1.6(10)
C5A	19.8(11)	17.3(14)	21.3(11)	2.2(10)	4.1(9)	-1.4(9)
C4A	22.6(12)	25.2(16)	25.7(12)	-5.6(11)	1.0(9)	4.1(10)
C7A	24.6(12)	15.5(13)	23.1(11)	-0.9(10)	0.4(9)	-3.2(10)
C6A	22.8(12)	17.4(14)	22.4(11)	0.6(10)	1.9(9)	-2.2(10)
C2B	24.3(12)	15.9(13)	21.1(11)	0.8(9)	0.3(9)	-1.6(10)
C12B	26.8(12)	19.8(15)	22.2(11)	4.4(10)	-2.1(9)	2.2(10)
C7B	25.7(12)	17.8(13)	20.2(11)	1.5(10)	-1.9(9)	-2.6(10)
C6B	21.8(12)	20.0(14)	25.5(11)	3.6(10)	-0.4(9)	-2.9(10)
C2A	21.0(12)	20.9(14)	20.9(11)	2.7(10)	1.6(9)	-2.1(10)
C8B	23.5(12)	15.2(14)	18.0(11)	-0.9(9)	2.3(9)	-0.4(9)
C5B	18.8(11)	17.1(13)	24.1(11)	4.2(10)	3.3(9)	-2.0(10)
C3A	27.1(13)	23.4(15)	24.7(12)	-5.8(10)	0.4(9)	2.7(11)
C3B	24.5(12)	21.2(14)	19.9(10)	0.7(10)	4.0(9)	0.6(10)
C10A	38.0(14)	23.3(15)	22.3(11)	4.3(11)	6.0(10)	-3.7(12)

Table 3 Anisotropic Displacement Parameters (Å^2×10^3) for lub1421. The Anisotropic displacement factor exponent takes the form: $-2\pi^2[h^2a^{*2}U_{11}+2hka^*b^*U_{12}+\ldots]$.

Atom	U_{11}	U_{22}	U_{33}	U_{23}	U_{13}	U_{12}
C14A	31.6(14)	27.1(16)	24.7(12)	1.0(11)	3.7(10)	-0.5(11)
C14B	32.3(14)	31.0(16)	25.5(12)	6.1(11)	3.8(10)	6.5(12)
C10B	36.7(14)	21.6(15)	23.5(11)	3.5(10)	7.4(10)	-3.3(12)
C15A	27.3(13)	38.5(19)	26.0(12)	5.2(12)	0.5(10)	5.6(12)
C15B	38.6(16)	31.0(17)	30.8(13)	-3.4(13)	-10.1(11)	4.8(13)
C13B	27.2(13)	22.6(15)	27.4(12)	1.5(11)	1.8(10)	1.2(11)
C13A	44.5(17)	24.4(17)	34.2(14)	-0.3(12)	7.6(12)	1.8(13)

Table 4 Bond Lengths for lub1421.

Atom	Atom	Length/Å	Atom	Atom	Length/Å
O1A	C1A	1.239(3)	N2A	C11A	1.353(3)
O4B	C9B	1.197(3)	N2A	C2A	1.445(3)
O2A	C6A	1.421(3)	C9B	C8B	1.512(4)
O1B	C1B	1.231(3)	C1A	C2A	1.523(3)
O6B	C11B	1.216(3)	C9A	C8A	1.519(3)
O4A	C9A	1.204(3)	C1B	C2B	1.528(3)
O3B	C9B	1.351(3)	C8A	C7A	1.534(3)
O3B	C10B	1.441(3)	C4B	C5B	1.517(3)
O5A	C11A	1.358(3)	C4B	C3B	1.553(3)
O5A	C12A	1.469(3)	C12A	C14A	1.519(3)
O5B	C11B	1.349(3)	C12A	C15A	1.516(4)
O5B	C12B	1.477(3)	C12A	C13A	1.510(4)
O3A	C9A	1.339(3)	C5A	C4A	1.531(4)
O3A	C10A	1.445(3)	C5A	C6A	1.552(3)
O2B	C6B	1.426(3)	C4A	C3A	1.545(3)
O6A	C11A	1.207(3)	C7A	C6A	1.524(3)
N1B	C1B	1.342(3)	C2B	C3B	1.540(3)
N1B	C8B	1.455(3)	C12B	C14B	1.520(4)
N1B	C5B	1.472(3)	C12B	C15B	1.525(4)
N1A	C1A	1.334(3)	C12B	C13B	1.515(4)
N1A	C8A	1.451(3)	C7B	C6B	1.526(4)
N1A	C5A	1.476(3)	C7B	C8B	1.542(3)
N2B	C11B	1.353(4)	C6B	C5B	1.532(3)
N2B	C2B	1.445(3)	C2A	C3A	1.533(4)

Table 5 Bond Angles for lub1421.

Atom	Atom	Atom	Angle/°	Atom	Atom	Atom	Angle/°
C9B	O3B	C10B	115.9(2)	O5A	C12A	C13A	109.3(2)
C11A	O5A	C12A	119.7(2)	C15A	C12A	C14A	110.9(2)
C11B	O5B	C12B	120.5(2)	C13A	C12A	C14A	111.0(2)
C9A	O3A	C10A	114.6(2)	C13A	C12A	C15A	111.1(2)
C1B	N1B	C8B	123.7(2)	N1A	C5A	C4A	110.1(2)
C1B	N1B	C5B	122.43(19)	N1A	C5A	C6A	103.23(18)
C8B	N1B	C5B	113.05(18)	C4A	C5A	C6A	116.6(2)
C1A	N1A	C8A	123.31(19)	C5A	C4A	C3A	112.5(2)
C1A	N1A	C5A	122.81(19)	C6A	C7A	C8A	104.40(19)
C8A	N1A	C5A	113.47(18)	O2A	C6A	C5A	113.5(2)
C11B	N2B	C2B	122.2(2)	O2A	C6A	C7A	107.89(19)
C11A	N2A	C2A	120.2(2)	C7A	C6A	C5A	103.68(19)
O6B	C11B	O5B	125.4(2)	N2B	C2B	C1B	112.1(2)
O6B	C11B	N2B	125.0(2)	N2B	C2B	C3B	110.6(2)
O5B	C11B	N2B	109.5(2)	C1B	C2B	C3B	113.6(2)
O4B	C9B	O3B	123.9(2)	O5B	C12B	C14B	110.1(2)
O4B	C9B	C8B	127.7(2)	O5B	C12B	C15B	102.4(2)
O3B	C9B	C8B	108.4(2)	O5B	C12B	C13B	110.62(19)
O1A	C1A	N1A	124.8(2)	C14B	C12B	C15B	110.0(2)
O1A	C1A	C2A	122.7(2)	C13B	C12B	C14B	112.9(2)
N1A	C1A	C2A	112.50(19)	C13B	C12B	C15B	110.3(2)
O4A	C9A	O3A	123.9(2)	C6B	C7B	C8B	103.95(19)
O4A	C9A	C8A	125.2(2)	O2B	C6B	C7B	107.2(2)
O3A	C9A	C8A	110.9(2)	O2B	C6B	C5B	113.9(2)
O1B	C1B	N1B	124.1(2)	C7B	C6B	C5B	101.8(2)
O1B	C1B	C2B	123.3(2)	N2A	C2A	C1A	109.12(19)
N1B	C1B	C2B	112.4(2)	N2A	C2A	C3A	112.7(2)
N1A	C8A	C9A	109.7(2)	C1A	C2A	C3A	109.1(2)
N1A	C8A	C7A	102.01(18)	N1B	C8B	C9B	112.8(2)
C9A	C8A	C7A	115.0(2)	N1B	C8B	C7B	102.06(18)
C5B	C4B	C3B	110.4(2)	C9B	C8B	C7B	113.6(2)
O6A	C11A	O5A	126.2(2)	N1B	C5B	C4B	108.2(2)
O6A	C11A	N2A	124.4(2)	N1B	C5B	C6B	102.89(18)
N2A	C11A	O5A	109.3(2)	C4B	C5B	C6B	118.1(2)
O5A	C12A	C14A	112.2(2)	C2A	C3A	C4A	112.4(2)
O5A	C12A	C15A	102.1(2)	C2B	C3B	C4B	113.25(19)

Table 6 Hydrogen Bonds for lub1421.

D	H	A	d(D-H)/Å	d(H-A)/Å	d(D-A)/Å	D-H-A/°
N2B	H2BA	O6B[1]	0.83(3)	2.33(3)	3.065(3)	147(3)
N2A	H2AA	O1A	0.83(3)	2.26(3)	2.686(3)	112(3)
O2A	H2A	O1B	0.92(3)	1.80(3)	2.707(2)	166(4)
O2B	H2B	O1A[2]	0.93(3)	1.88(3)	2.805(3)	172(4)

[1]+X,1+Y,+Z; [2]-1+X,1+Y,+Z

Table 7 Torsion Angles for lub1421.

A	B	C	D	Angle/°	A	B	C	D	Angle/°
O1A	C1A	C2A	N2A	3.2(4)	C8A	C7A	C6A	O2A	85.2(2)
O1A	C1A	C2A	C3A	126.7(3)	C8A	C7A	C6A	C5A	-35.4(2)
O4B	C9B	C8B	N1B	-1.2(4)	C11A	O5A	C12A	C14A	-68.3(3)
O4B	C9B	C8B	C7B	114.4(3)	C11A	O5A	C12A	C15A	173.0(2)
O1B	C1B	C2B	N2B	10.3(4)	C11A	O5A	C12A	C13A	55.3(3)
O1B	C1B	C2B	C3B	136.7(3)	C11A	N2A	C2A	C1A	-149.8(3)
O4A	C9A	C8A	N1A	-1.0(3)	C11A	N2A	C2A	C3A	88.9(3)
O4A	C9A	C8A	C7A	113.3(3)	C12A	O5A	C11A	O6A	23.6(4)
O3B	C9B	C8B	N1B	177.18(19)	C12A	O5A	C11A	N2A	-159.2(2)
O3B	C9B	C8B	C7B	-67.2(2)	C5A	N1A	C1A	O1A	-177.7(2)
O3A	C9A	C8A	N1A	177.32(19)	C5A	N1A	C1A	C2A	1.0(3)
O3A	C9A	C8A	C7A	-68.4(3)	C5A	N1A	C8A	C9A	101.5(2)
O2B	C6B	C5B	N1B	-81.6(3)	C5A	N1A	C8A	C7A	-20.8(3)
O2B	C6B	C5B	C4B	37.4(3)	C5A	C4A	C3A	C2A	-7.4(3)
N1B	C1B	C2B	N2B	-172.5(2)	C4A	C5A	C6A	O2A	26.5(3)
N1B	C1B	C2B	C3B	-46.2(3)	C4A	C5A	C6A	C7A	143.3(2)
N1A	C1A	C2A	N2A	-175.5(2)	C6A	C5A	C4A	C3A	-157.1(2)
N1A	C1A	C2A	C3A	-52.0(3)	C2B	N2B	C11B	O6B	-3.6(4)
N1A	C8A	C7A	C6A	34.3(2)	C2B	N2B	C11B	O5B	177.9(2)
N1A	C5A	C4A	C3A	-40.0(3)	C12B	O5B	C11B	O6B	-2.0(4)
N1A	C5A	C6A	O2A	-94.4(2)	C12B	O5B	C11B	N2B	176.4(2)
N1A	C5A	C6A	C7A	22.4(2)	C7B	C6B	C5B	N1B	33.4(3)
N2B	C2B	C3B	C4B	161.7(2)	C7B	C6B	C5B	C4B	152.4(2)
N2A	C2A	C3A	C4A	175.2(2)	C6B	C7B	C8B	N1B	31.5(2)
C11B	O5B	C12B	C14B	-61.8(3)	C6B	C7B	C8B	C9B	-90.3(2)
C11B	O5B	C12B	C15B	-178.8(2)	C2A	N2A	C11A	O5A	175.8(2)
C11B	O5B	C12B	C13B	63.7(3)	C2A	N2A	C11A	O6A	-6.9(5)
C11B	N2B	C2B	C1B	-105.3(3)	C8B	N1B	C1B	O1B	11.2(4)
C11B	N2B	C2B	C3B	126.7(3)	C8B	N1B	C1B	C2B	-165.9(2)

Table 7 Torsion Angles for lub1421.

A	B	C	D	Angle/°	A	B	C	D	Angle/°
C1A	N1A	C8A	C9A	-71.3(3)	C8B	N1B	C5B	C4B	-140.3(2)
C1A	N1A	C8A	C7A	166.4(2)	C8B	N1B	C5B	C6B	-14.7(3)
C1A	N1A	C5A	C4A	46.9(3)	C8B	C7B	C6B	O2B	79.3(2)
C1A	N1A	C5A	C6A	172.0(2)	C8B	C7B	C6B	C5B	-40.6(2)
C1A	C2A	C3A	C4A	53.8(3)	C5B	N1B	C1B	O1B	179.9(2)
C9A	C8A	C7A	C6A	-84.4(2)	C5B	N1B	C1B	C2B	2.8(3)
C1B	N1B	C8B	C9B	-78.4(3)	C5B	N1B	C8B	C9B	112.0(2)
C1B	N1B	C8B	C7B	159.3(2)	C5B	N1B	C8B	C7B	-10.3(3)
C1B	N1B	C5B	C4B	49.9(3)	C5B	C4B	C3B	C2B	16.5(3)
C1B	N1B	C5B	C6B	175.6(2)	C3B	C4B	C5B	N1B	-56.6(3)
C1B	C2B	C3B	C4B	34.5(3)	C3B	C4B	C5B	C6B	-172.8(2)
C8A	N1A	C1A	O1A	-5.5(4)	C10A	O3A	C9A	O4A	1.1(3)
C8A	N1A	C1A	C2A	173.1(2)	C10A	O3A	C9A	C8A	-177.2(2)
C8A	N1A	C5A	C4A	-126.0(2)	C10B	O3B	C9B	O4B	-0.4(4)
C8A	N1A	C5A	C6A	-0.8(3)	C10B	O3B	C9B	C8B	-178.8(2)

Table 8 Hydrogen Atom Coordinates (Å×10^4) and Isotropic Displacement Parameters (Å^2×10^3) for lub1421.

Atom	*x*	*y*	*z*	U(eq)
H8A	6239.25	270.6	5545.35	23
H4BA	-1376.38	7282.16	6230.66	27
H4BB	-549.4	9257.37	6139.45	27
H5A	4573.38	726.23	6122.17	23
H4AA	4114.94	4456.03	6305.87	29
H4AB	4602.29	6007.55	6067.62	29
H7AA	4398.15	1255.25	5340.54	25
H7AB	4551.68	-1028.44	5547.83	25
H6A	3346.87	1227.61	5775.02	25
H2BB	779.71	3364.21	6310.28	25
H7BA	-443.64	6592.13	5255.51	26
H7BB	-382.64	3956.39	5405.9	26
H6B	-1735.22	5858.87	5626.53	27
H2AB	6107.62	1179.64	6437.88	25
H8B	1286.8	5146.35	5505.79	23
H5B	-617.82	4176.23	5960.6	24
H3AA	6081.71	6391.69	6310.36	30
H3AB	5539.9	5172.04	6562.42	30
H3BA	363.07	8145.31	6522.03	26

Table 8 Hydrogen Atom Coordinates (Å×10^4) and Isotropic Displacement Parameters (Å^2×10^3) for lub1421.

Atom	*x*	*y*	*z*	U(eq)
H3BB	-288.87	5681.9	6554.72	26
H10A	6374.44	5821.42	4851.3	42
H10B	7144.83	6446.39	5105.7	42
H10C	5995.74	7472.79	5096.47	42
H14A	8598.73	4297.52	7408.3	42
H14B	8762.57	1670.46	7551.02	42
H14C	7726.12	2238.64	7374.51	42
H14D	2508.48	1060.71	7280.92	44
H14E	3241.47	-1295.14	7275.13	44
H14F	2358.9	-917.08	7040.78	44
H10D	1850.96	11005.95	4894.88	41
H10E	2344.96	11746.61	5188.38	41
H10F	1196.57	12555.37	5101.73	41
H15A	10409.41	1603.72	6968.64	46
H15B	10470.36	1432.51	7294.41	46
H15C	10197.73	3994.41	7147.44	46
H15D	4847.79	3804.39	7072.35	51
H15E	4804.45	1656.59	7294.35	51
H15F	4019	3911.96	7301	51
H13D	3703.54	-1105.05	6668.69	39
H13E	4600.66	-1501.31	6898.69	39
H13F	4657.9	742.4	6689.03	39
H13A	8066.14	-1622.85	7106.63	51
H13B	9177.75	-2108.67	7248.39	51
H13C	9045.12	-1789.62	6924.53	51
H2BA	2120(30)	6440(60)	6595(7)	41
H2AA	7960(20)	3080(80)	6414(6)	41
H2A	3290(30)	5360(70)	5748(7)	41
H2B	-1620(30)	10060(80)	5731(7)	41

Experimental

Single crystals of $C_{15}H_{24}N_2O_6$ **[lub1421]** were **[]**. A suitable crystal was selected and **[mounted on a Mitegen microloop (M8-50V)]** on a **Bruker Smart APEX** diffractometer. The crystal was kept at 100 K during data collection. Using Olex2 [1], the structure was solved with the XT [2] structure solution program using Intrinsic Phasing and refined with the XL [3] refinement package using Least Squares minimisation.

1. Dolomanov, O.V., Bourhis, L.J., Gildea, R.J, Howard, J.A.K. & Puschmann, H. (2009), J. Appl. Cryst. 42, 339-341.
2. Sheldrick, G.M. (2015). Acta Cryst. A71, 3-8.
3. Sheldrick, G.M. (2008). Acta Cryst. A64, 112-122.

Crystal structure determination of [lub1421]

Crystal Data for $C_{15}H_{24}N_2O_6$ (*M* =328.36 g/mol): monoclinic, space group I2 (no. 5), a = 12.8006(6) Å, b = 5.3251(2) Å, c = 48.103(2) Å, β = 92.926(2)°, V = 3274.6(2) Å^3, Z = 8, T = 100 K, μ(Cu Kα) = 0.863 mm^{-1}, *Dcalc* = 1.332 g/cm^3, 29804 reflections measured (3.678° ≤ 2Θ ≤ 143.742°), 5590 unique (R_{int} = 0.0479, R_{sigma} = 0.0375) which were used in all calculations. The final R_1 was 0.0355 (I > 2σ(I)) and wR_2 was 0.0897 (all data).

Refinement model description

Number of restraints - 3, number of constraints - unknown.

Details:

```
1. Twinned data refinement
 Scales: 0.95(19)
 0.05(19)
2. Fixed Uiso
 At 1.2 times of:
  All C(H) groups, All C(H,H) groups, All C(H,H,H,H) groups
 At 1.5 times of:
  All C(H,H,H) groups
3. Restrained distances
 H2A-O2A ≈ H2B-O2B
 with sigma of 0.02
 H2BA-N2B ≈ H2AA-N2A
 with sigma of 0.02
4.a Ternary CH refined with riding coordinates:
 C8A(H8A), C5A(H5A), C6A(H6A), C2B(H2BB), C6B(H6B), C2A(H2AB), C8B(H8B),
 C5B(H5B)
4.b Secondary CH2 refined with riding coordinates:
 C4B(H4BA,H4BB), C4A(H4AA,H4AB), C7A(H7AA,H7AB), C7B(H7BA,H7BB), C3A(H3AA,
 H3AB), C3B(H3BA,H3BB)
4.c Idealised Me refined as rotating group:
 C10A(H10A,H10B,H10C), C14A(H14A,H14B,H14C), C14B(H14D,H14E,H14F), C10B(H10D,
 H10E,H10F), C15A(H15A,H15B,H15C), C15B(H15D,H15E,H15F), C13B(H13D,H13E,H13F),
 C13A(H13A,H13B,H13C)
```
